# Identification of histone deacetylase inhibitors with (arylidene)aminoxy scaffold active in uveal melanoma cell lines

**DOI:** 10.1080/14756366.2020.1835883

**Published:** 2020-10-26

**Authors:** Susanna Nencetti, Doretta Cuffaro, Elisa Nuti, Lidia Ciccone, Armando Rossello, Marina Fabbi, Flavio Ballante, Gabriella Ortore, Grazia Carbotti, Francesco Campelli, Irene Banti, Rosaria Gangemi, Garland R. Marshall, Elisabetta Orlandini

**Affiliations:** a Dipartimento di Farmacia, Università di Pisa, Pisa, Italy; b Research Center “E. Piaggio”, Università di Pisa, Pisa, Italy; c IRCCS Ospedale Policlinico San Martino, Genova, Italy; d Department of Biochemistry and Molecular Biophysics, Washington University School of Medicine, St. Louis, MO, USA; e Dipartimento di Scienze della Terra, Università di Pisa, Pisa, Italy

**Keywords:** Uveal melanoma, HDAC inhibitors, HDAC6, SAHA, (arylidene)aminoxy-based hydroxamates

## Abstract

Uveal melanoma (UM) represents an aggressive type of cancer and currently, there is no effective treatment for this metastatic disease. In the last years, histone deacetylase inhibitors (HDACIs) have been studied as a possible therapeutic treatment for UM, alone or in association with other chemotherapeutic agents. Here we synthesised a series of new HDACIs based on the SAHA scaffold bearing an (arylidene)aminoxy moiety. Their HDAC inhibitory activity was evaluated on isolated human HDAC1, 3, 6, and 8 by fluorometric assay and their binding mode in the catalytic site of HDACs was studied by molecular docking. The most promising hit was the quinoline derivative **VS13**, a nanomolar inhibitor of HDAC6, which exhibited a good antiproliferative effect on UM cell lines at micromolar concentration and a capability to modify the mRNA levels of HDAC target genes similar to that of SAHA.

## 1. Introduction

Uveal melanoma (UM) is a highly aggressive form of melanoma and is the most common primary intraocular tumour in adults. The traditional clinical treatment of patients with UM is the enucleation of the affected eye or, to preserve the vision or the globe, other clinical options are radiotherapy, photodynamic therapy, and systemic chemotherapy^1^. About 50% of the patients have strong tendency to develop lethal metastasis, principally to the liver (89%), that are usually identified 2–5 years after treatment of the primary tumour and are poorly sensitive to chemotherapy. Thus, the prognosis of these patients becomes poor with a median survival of 2–18 months. UM can be grouped in two classes on the basis of their gene expression profile: Class I tumours with very low metastatic risk and Class II with a high risk of metastasis^2^. In the last 10 years metastatic UM has been studied at the genetic and molecular levels to discover effective target therapies. Epigenetic alterations, such as changes in DNA methylation and histone acetylation status, are involved in tumour progression^3^.

In the past years, a group of enzymes involved in the epigenetic regulation of gene expression, histone deacetylases (HDACs), have generated increasing interest as potential therapeutic targets for UM^4–6^. HDACs remove the acetyl groups from histone lysine residues from diverse protein targets, resulting in a condensed chromatin structure that downregulates gene expression, also of tumour suppressor genes^7^. The status of histone acetylation depends on the balance between histone acetylation and deacetylation, induced by histone acetyltransferases (HATs), and HDACs, respectively. Increasing evidence suggests that the alteration of HAT/HDAC activity is present in cancer^8^. In mammalians, 18 different HDACs have been identified and divided into four classes (Class I, Class II, Class III, and Class IV) based on their sequence homology to yeast proteins domain organisation and subcellular localisation. Class I (subtypes 1,2,3,8), II (subtypes 4,5,6,7,9,10), and IV (subtype 11)^9^ HDACs are zinc-dependent enzymes, while Class III HDACs (SIRTs 1–7) require the cofactor NAD^+^ to express their activity. Class I can generally be detected in the nucleus and is ubiquitously expressed.

HDACs are overexpressed in a wide range of diseases including tumours and consequently become attractive targets for the development of drugs for cancer treatment^10–12^.

HDAC inhibitors (HDACIs) interfere with the deacetylation process mediated by HDACs with an increase of histone acetylation. HDACIs induce cancer cell death through several pathways: apoptosis, differentiation, cell cycle arrest, and suppression of cell migration. In UM HDACIs induce morphological differentiation and cell-cycle arrest and inhibit the growth of UM tumour *in vivo*^5^^,^^13^^,^^14^.

**Figure 1.**
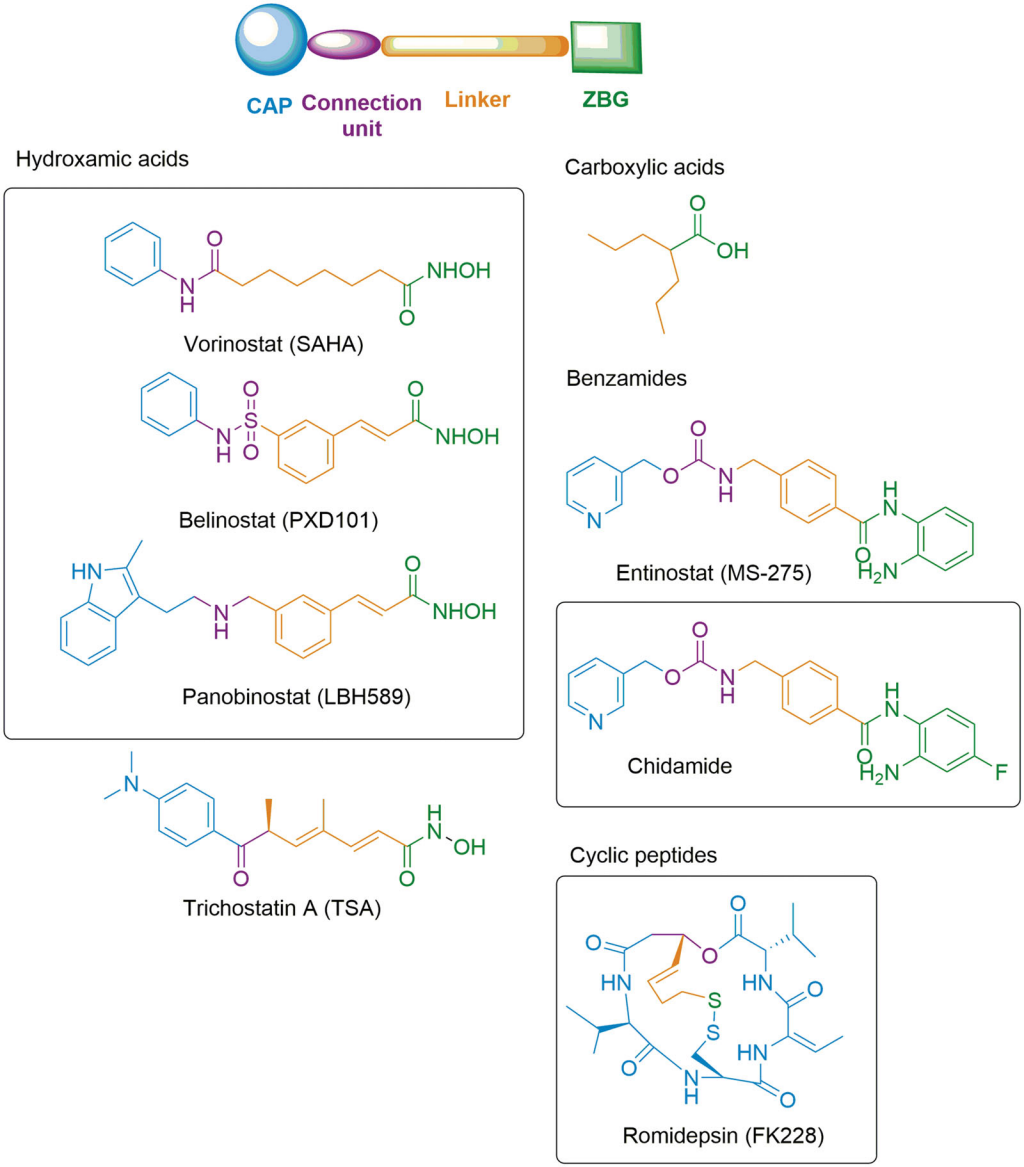
Representative compounds of the different structural classes of HDAC inhibitors. Chemical structures of clinically approved drugs are highlighted in box.

The first HDAC inhibitor approved by the FDA, in 2006, was Vorinostat (SAHA) for the treatment of cutaneous T-cell lymphoma (CTCL)^15^. To date other three HDAC inhibitors have been approved by FDA: Romidepsin (FK228)^16^ in 2009, Belinostat (PXD101)^17^ in 2014 for the treatment of CTLC or Peripheral T-cell lymphoma (PTCL), and Panobinostat (LBH589)^18^ in 2015 for Multiple myeloma and CTCL. Chidamide (HBI-8000) has been approved by the Chinese FDA for relapsed or refractory PTCL in 2015^19^ (Figure 1). *In vitro* studies with some of these HDACIs have shown a shift of the gene expression profile from Class II (high metastatic risk) to a Class I (low metastatic risk) in UM cell cultures^5^. Moreover, it has been recognised that HDAC inhibitors such as Vorinostat (SAHA) and Panobinostat (LBH589), may be useful in adjuvant therapy in patients with high-risk Class II UM playing a role in preventing the progression of micrometastatic disease^13^.

**Figure 2.**
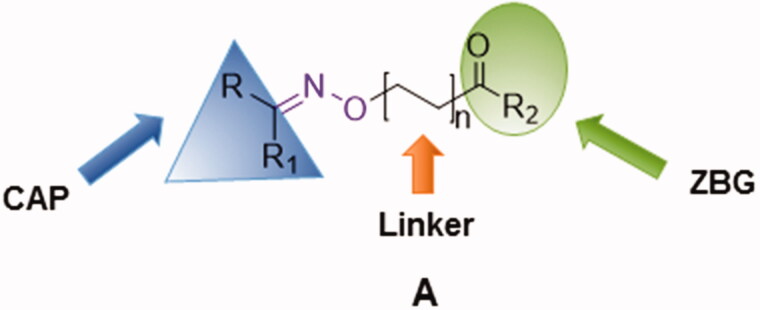
Pharmacophore model of the newly synthesised compounds.

Several structural classes of HDAC inhibitors, both natural and synthetic, have been reported in the literature such as hydroxamates, carboxylates, benzamides, and cyclic peptides (Figure 1). A variety of derivatives of each class have been synthesised and characterised^20^. All classes of HDAC inhibitors share a common pharmacophore model, which consists of a CAP group, a connection unit (CU), a linker and a zinc-binding group (ZBG) that is mostly represented by a hydroxamic acid (Figure 2). Modifications of these four structural parts such as the CAP group, the linker, or the ZBG, have become important strategies that have led to the development of a large number of HDACIs^21^.

As part of our ongoing efforts in identifying new HDAC inhibitors potentially active in UM, we decided to develop a series of hydroxamates based on SAHA scaffold. The choice of SAHA structure as a starting point of our study was based on two considerations: SAHA is an anticancer drug in clinical use and, moreover, its activity on UM has been already proved by Landreville *et al.* through an *in vitro* study on UM cell lines^13^.

**Scheme 1.**
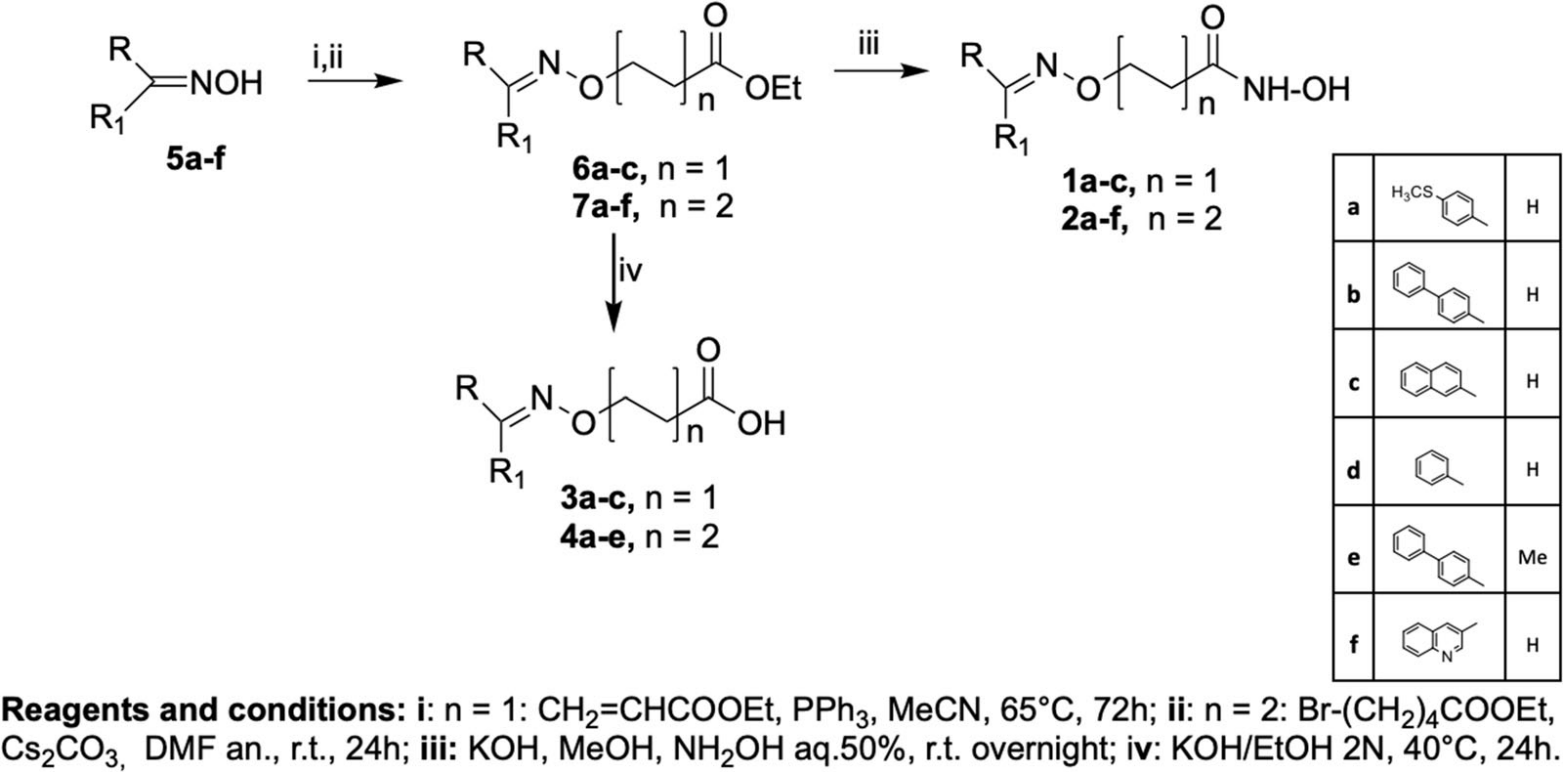
Synthesis of the hydroxamic acids 1a-c, 2a-f and carboxylic acids 3a-c, 4a-e.

In this context, we envisioned developing new HDAC SAHA-based inhibitors of general structure **A** (Figure 2) in which the amidic group of SAHA is replaced by a different connection unit (CU). In fact, in the synthesised compounds (**1a–c, 2a–f**, **3a–c** and **4a–e**, Scheme 1) the linker and the hydrophobic CAP group (R) are connected via an oxime ether moiety (C = N–O–) possessing a sp^2^-hybridization, analogously to other CU used in many HDAC inhibitors^22^.

Furthermore, since it is widely accepted that the CAP group plays an important role in the HDAC inhibition profile, we have synthesised new derivatives in which the CAP group was changed from a phenyl group of SAHA into a more bulky aromatic moiety. We have also investigated the influence of the linker length on HDAC inhibition activity synthesising compounds with two or four carbon atoms in the linear alkyl chain (Figure 2).

In the present paper, we report the synthesis and HDAC inhibitory activity of both hydroxamic compounds of type **A** (R_2_=NH–OH, **1a–c**, **2a–f**) and their corresponding carboxylic acid analogues (R_2_=OH, **3a–c**, **4a–e**). The HDAC inhibitory activity of the new compounds was first evaluated against HeLa cell nuclear extract containing a mixture of Class I HDACs. Then, with the aim to deeply investigate the biological properties of the four most promising hydroxamic derivatives, we performed *in vitro* assays on isolated Class I enzymes (HDACs 1,3,8) and Class II HDAC6. Considering the expression of histone deacetylases in UM^23^ and the potential therapeutic role of HDACIs in this tumour^5^, the same compounds were selected for further tests on UM cell lines. Finally, docking studies were performed to investigate their binding mode.

## 2. Results and discussion

### 2.1. Chemistry

The synthesis of the target compounds is outlined in Scheme 1. Ethyl esters **6a–c** were obtained by Michael–type reaction of oximes **5a–f** with ethyl acrylate, while ethyl esters **7a–f** were obtained by reaction of the opportune oximes **5a–f** with the ethyl ester of 5-bromovaleric acid using Cs_2_CO_3_ as base. The hydroxamic acids **1a–c** (*n* = 1) and **2a–f** (*n* = 2) were directly obtained from the ethyl esters **6a–c** and **7a–f**, respectively, by treatment with aqueous hydroxylamine in basic conditions. The carboxylic acids **3a–c** (*n* = 1) and **4a–d** (*n* = 2) were obtained by saponification with 2 N KOH in ethanol of the ethyl esters **6a–c** and **7a–f**.

### 2.2. In vitro HDACs inhibition assays

The hydroxamic acids **1a–c**, **2a–f** and carboxylic acids **3a–c**, **4a––e** synthesised in this study were preliminarily tested on a HeLa nuclear extract containing a mixture of HDACs, by using a commercially available HDAC assay kit (BIOMOL). Inhibitory data expressed as IC_50_ values are reported in Table 1. SAHA and Trichostatin A (TSA) were used as positive controls.

**Table 1.** HDACs inhibitory activity of the newly synthesised compounds **1a–c**, **2a–f**, **3a–c**, **4a–e**.
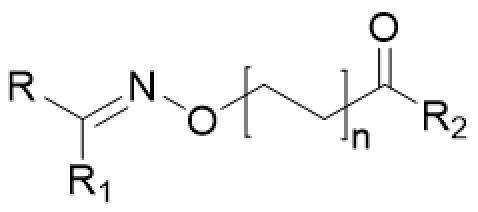

Compd.	*R*	*R*_1_	*R*_2_	*n*	IC_50_ on Hela extract (µM)^a^
**1a**		H	NHOH	1	NT^b^
**3a**		H	OH	1	>100
**2a**		H	NHOH	2	2.880
**4a**		H	OH	2	>100
**1b**		H	NHOH	1	43.2
**3b**		H	OH	1	>100
**2b (SN2)**		H	NHOH	2	0.056
**4b**		H	OH	2	>100
**1c**		H	NHOH	1	3.346
**3c**		H	OH	1	>100
**2c (LD10)**		H	NHOH	2	0.096
**4c**		H	OH	2	>100
**2d**		H	NHOH	2	4.6
**4d**		H	OH	2	>100
**2e (VS16)**		Me	NHOH	2	0.053
**4e**		Me	OH	2	>100
**2f (VS13)**		H	NHOH	2	0.054
**SAHA**					0.280
**TSA**					0.037

^a^ Values are means of at least three experiments, SD values are <20% of the mean; ^b^not tested.

The results of this preliminary screening, reported in Table 1, showed that compounds bearing a hydroxamic moiety as ZBG possess HDAC inhibitory activity while the carboxylic function as ZBG is detrimental to HDAC inhibition. The length of the linker influences the HDAC inhibitory activity and a 4-carbon linear aliphatic chain appears nearly optimal: IC_50_ values of compounds with *n* = 1 are in the micromolar range while the IC_50_ values of compounds with *n* = 2 are in the nanomolar range. Among the hydroxamic acids with *n* = 2, those having bulkier CAP groups exhibited superior activity than monoacrylic derivatives. The most potent compounds, **2b (SN2)**, **2c (LD10)**, **2e (VS16)**, and **2f (VS13)**, as shown in Table 1, resulted 3- to 5-folds more potent than SAHA and were selected for a deeper biological evaluation.

The selected hydroxamates (**SN2**, **VS16**, **LD10**, and **VS13**) were tested for their inhibitory activity towards four human isolated HDACs: HDACs 1, 3, and 8 (Class I HDACs) and HDAC 6 (a Class II HDAC) to investigate how the different CAP groups could influence the HDAC activity^24^. In particular, HDAC6, which was not present in the HeLa nuclear extract used for the preliminary tests, is considered an important target in cancer therapy^25–27^. SAHA, entinostat, and tubastatin A were used as positive controls. As summarised in Table 2, all compounds (except LD10 at HDAC1) were able to inhibit HDAC1, HDAC3, and HDAC6 in the nanomolar range while they were less efficient against HDAC8, displaying IC_50_ values in the low micromolar range. Moreover, compounds **SN2**, **VS16**, and **LD10** resulted in more active on HDAC3 than on the other isoforms. For example, **SN2** and **VS16** had lower IC_50_ values (58 nM and 43 nM, respectively) for HDAC3 than those for HDAC1 (698 nM and 593 nM, respectively), or for HDAC6 (316 nM and 406 nM, respectively). Dose-response curves are illustrated in Supplementary Figure S1.

**Table 2.** HDACs inhibitory selectivity profile of selected hydroxamic acids, SAHA, entinostat and tubastatin A.

Compd.	Chemical structure	IC_50_ (µM)^a^			
		HDAC1	HDAC3	HDAC6	HDAC8
**SN2**		0.698	0.058	0.316	8.206
**VS16**		0.593	0.043	0.406	13.680
**LD10**		6.043	0.594	0.836	>30
**VS13**		0.137	0.040	0.010	9.29
**SAHA**		0.007	0.0014	0.0014	0.495
**Entinostat**		1.480^b^	0.790^b^	>30^b^	>30^b^
**Tuastatin A**		2.866	0.766	0.015	2.341

^a^Compounds were tested in duplicate in a 10-point dose curve with 3-fold serial dilution starting from 30 μM. In the case of SAHA a 15-point dose curve on HDACs 3 and 6 was performed. ^b^See ref.^34^.

**Table 3.** QRT-PCR primers.

Gene	Forward primer	Reverse primer
*GAPDH*	GAAGGTGAAGGTCGGAGT	CATGGGTGGAATCATATTGGAA
*POLR2A*	GACAATGCAGAGAAGCTGG	GCAGGAAGACATCATCATCC
*RAD54L*	CCCTTTCTTCCATCACCTCGCT	GCCTTAGAGCTGTAACCAGGAG
*RAD51*	TCTCTGGCAGTGATGTCCTGGA	TAAAGGGCGGTGGCACTGTCTA
*CLU (TRPM2)*	TGCGGATGAAGGACCAGTGTGA	TTTCCTGGTCAACCTCTCAGCG
*DHRS2 (Hep27)*	GGTGCTGTCATCCTGGTCTCTT	CCAGCTCCAATGCCAGTGTTCT
*CDKN1A (P21)*	AGGTGGACCTGGAGACTCTCAG	TCCTCTTGGAGAAGATCAGCCG

Among the four selected compounds, the most active on all the HDACs considered in this work was the quinoline derivative **VS13** that showed a stronger effect on HDAC6 than on the other isoforms. In particular, **VS13** was active at 10 nM on HDAC6, only 7 times less active than SAHA and ∼900 times more selective for HDAC6 over HDAC8.

These compounds were chosen for further evaluation on UM cell lines and docking studies were carried out to explain their different selectivity profile.

### 2.3. Docking studies

Compounds **LD10**, **VS13**, **SN2**, and **VS16** were docked in HDAC1, HDAC3, HDAC6, and HDAC8.

**Figure 3.**
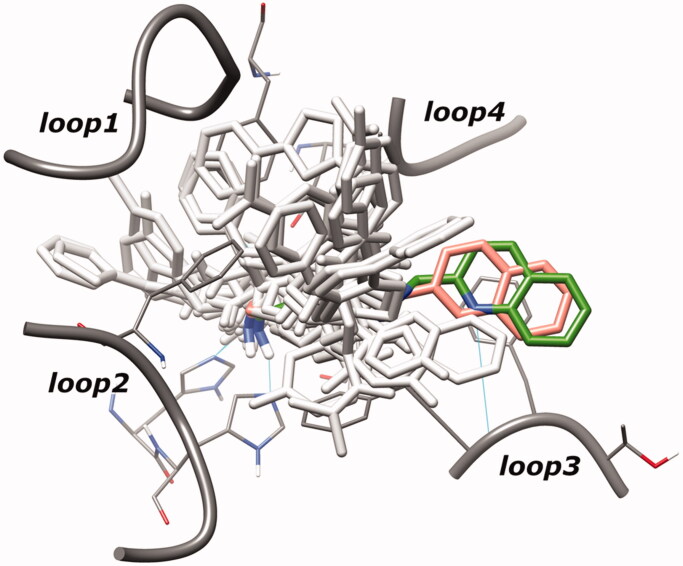
Superposition of hydroxamate inhibitors co-crystallised in human HDACs (light grey coloured) and docked-pose of inhibitors **LD10** and **VS13** (salmon and green coloured, respectively). For clarity, inhibitor poses extracted from different subtypes are shown in HDAC6 binding site.

The superposition between the docked-pose of two investigated compounds **LD10** and **VS13** and the PDB structures (human HDACs in complex with hydroxamate inhibitors^28^) showed a quite different disposition of both ligands in the HDACs binding site. Specifically, the CAP group of these inhibitors occupies a region between loop3 and loop4 of HDACs, while that of the crystallised hydroxamate inhibitors^28^ is allocated around loop1, loop2, and loop4 (Figure 3).

In the HDAC-inhibitor crystal complexes, the region filled by the CAP of our inhibitors is frequently (but not constantly) occupied by water molecules which engaged hydrogen bonds with the catalytic histidine. The different pose displayed by compounds **LD10** and **VS13** could be related to the substitution of the typical amidic connection unit, present in the HDAC inhibitors, with the methyloximino moiety. This group seems to modify the flexibility of the inhibitors and, consequently, the CAP fitting with the protein surface.

**Figure 4.**
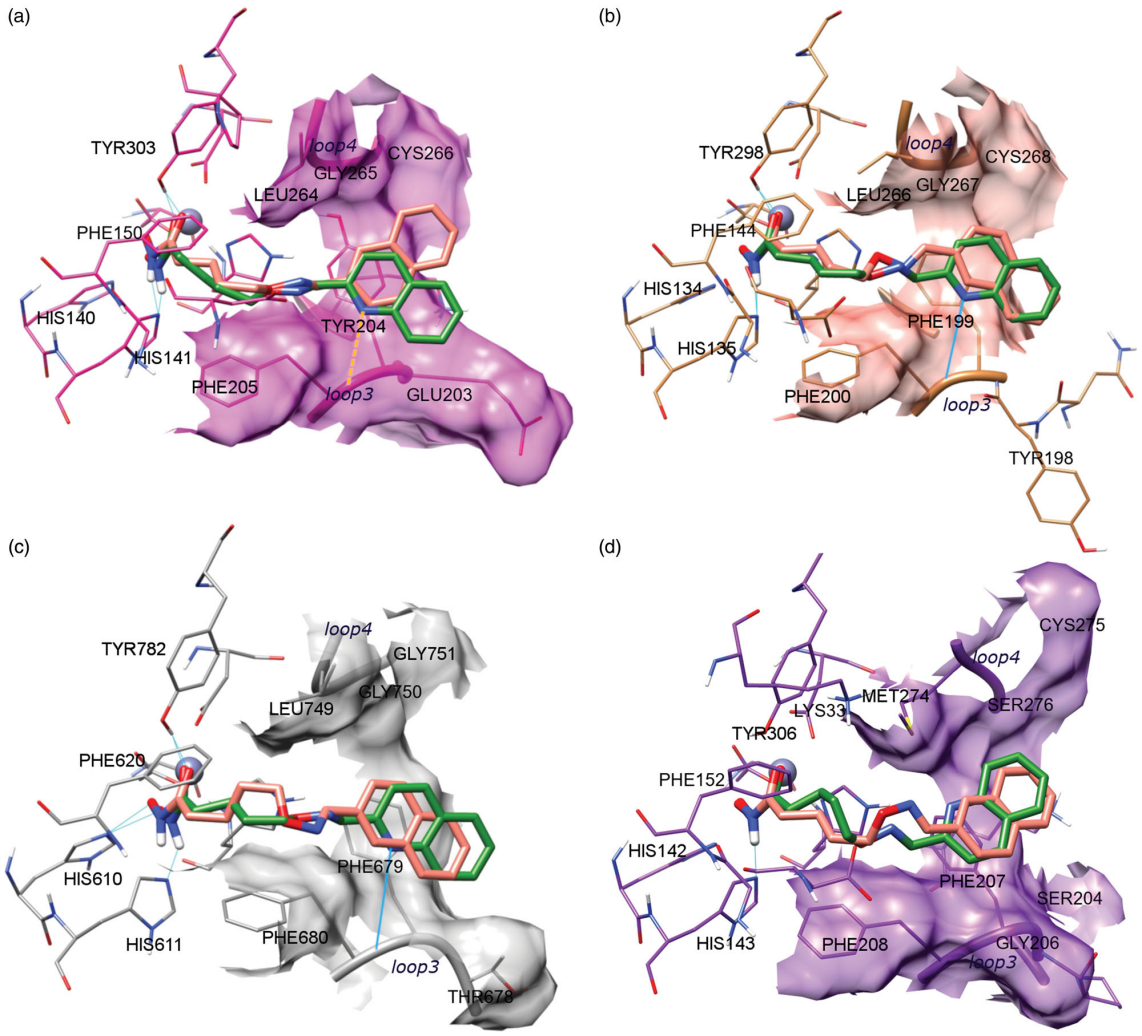
Docking of **LD10** (salmon) and **VS13** (green) in (a) HDAC1; (b) HDAC3; (c) HDAC6, and (d) HDAC8. Proper hydrogen bonds are highlighted in cyan.

**Figure 5.**
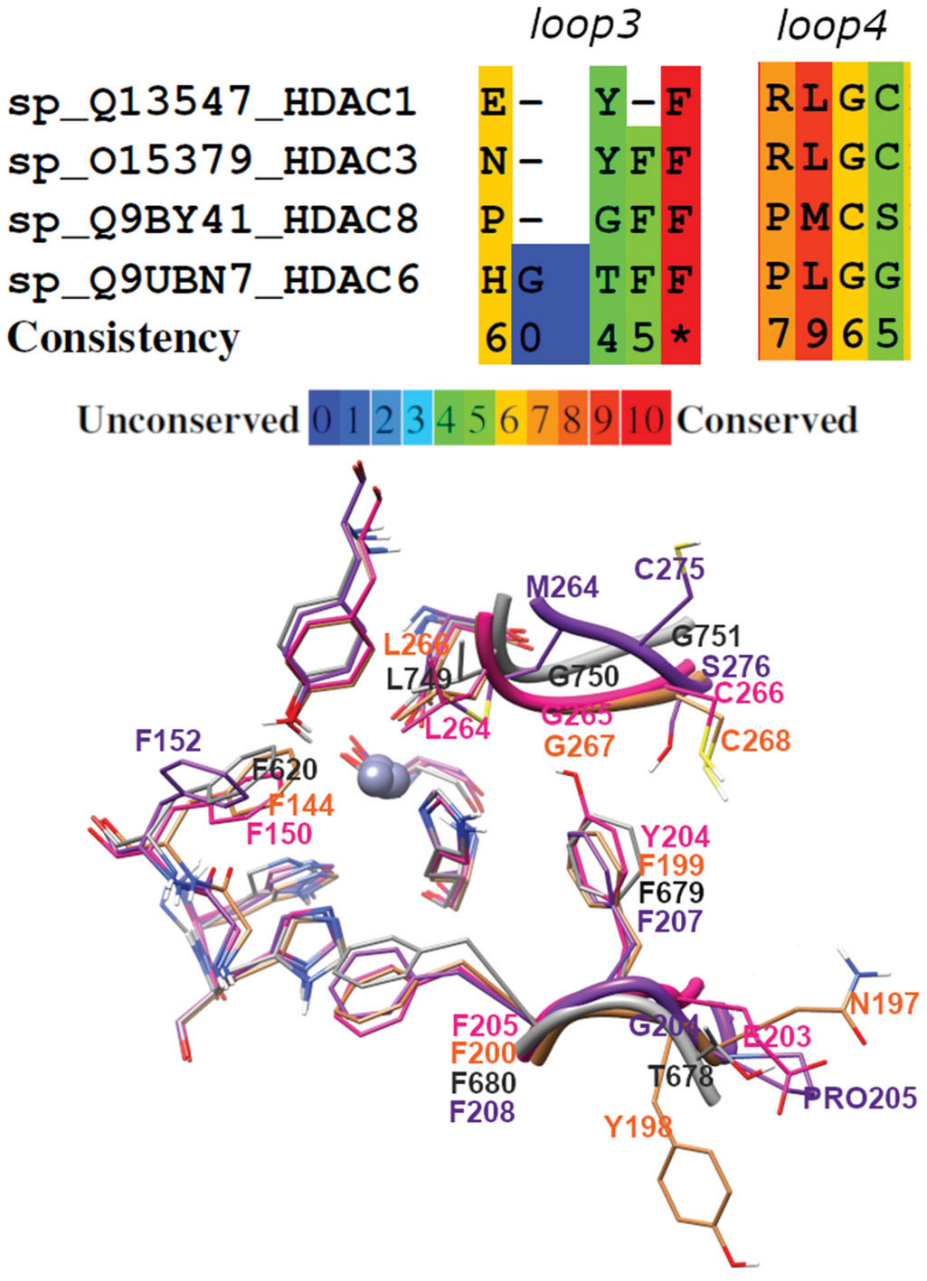
Sequence alignment of loop3 and loop4 regions of HDAC1, 3, 6, and 8. At bottom, superposition of aligned loop3 and loop4 crystal structures of the four HDAC subtypes (HDAC1, HADC3, HDAC6, and HDAC8 are magenta, salmon, grey, and purple coloured, respectively).

The comparison of the four HDAC subtypes highlighted that the core structure of the catalytic domain is well conserved (Figure 4(a–d)). The only exception was the different orientation displayed by Phe152, in HDAC8, with respect to Phe150 (HDAC1), Phe144 (HDAC3), and Phe620 (HDAC6) (Figure 4(a–d), Figure 5). Moreover, analysis of loops3 and 4 displayed a detectable sequence and conformational variability among the four HDAC isozymes (Figures 4 and 5).

The major differences were found in HDAC8 where both loops possess not conserved residues (Figure 5; alignment generated using PRALINE^29^) which influence the binding site surface and the backbone shape. Precisely, loop4 of HDAC8 opens the binding cavity outside, with respect to the arrangement of the same loop in HDAC1 and HDAC3 (see also the surface in Figure 4). On the contrary, the arrangement of loop3 in HDAC8 (as in HDAC1) shrinks the binding channel.

GOLD calculation showed that all ligands have a zinc-coordinated pose in the HDACs binding site.

Regarding the CAP group orientation, the differences between loop3 and 4 regions among the HDAC subtypes can induce a different pose of the studied ligands. While in HDAC3 and 6 the CAP group of **LD10** and **VS13** was oriented towards loop3, in HDAC8 it pointed towards loop4 (Figure 4). A similar trend to HDAC8 was registered in HDAC1 for **LD10** inhibitor.

In HDAC8, both ligands **LD10** and **VS13** coordinated the catalytic zinc and established one polar interaction with the His143 of the second binding shell (Figure 4(d)).

In HDAC1 core, the hydroxamic moiety of both inhibitors established two hydrogen bonds with His141 and Tyr303. In addition, the aromatic portion of **VS13** was orientated towards loop3 thanks to a weak polar interaction of the quinoline nitrogen with the backbone (d_H-_*_N_* = 2.8 Å) (Figure 4(a)).

In HDAC3, the **LD10** and **VS13** hydroxamate group was stabilised through two hydrogen interactions with His135 and Tyr298. Both aromatic CAP groups pointed towards loop3, in particular, the quinoline nitrogen of **VS13** engaged a strong hydrogen bond with the loop3’s backbone (Figure 4(b)).

A similar ligands pose was calculated in HDAC6 where the hydroxamate group was further stabilised by the His610 of the second catalytic shell (Figure 4(c)).

Therefore, docking results showed a progressive increment from HDAC8 to HDAC1, HDAC3 and HDAC6 in the polar interactions which strengthened the stabilisation of the inhibitors in the binding site and promoted the zinc coordination. Analysing the **VS13** binding pose, it can be hypothesised that a nitrogen atom in the aromatic CAP ring allows better interaction with loop3 and, therefore, enhances the inhibitory activity.

**Figure 6.**
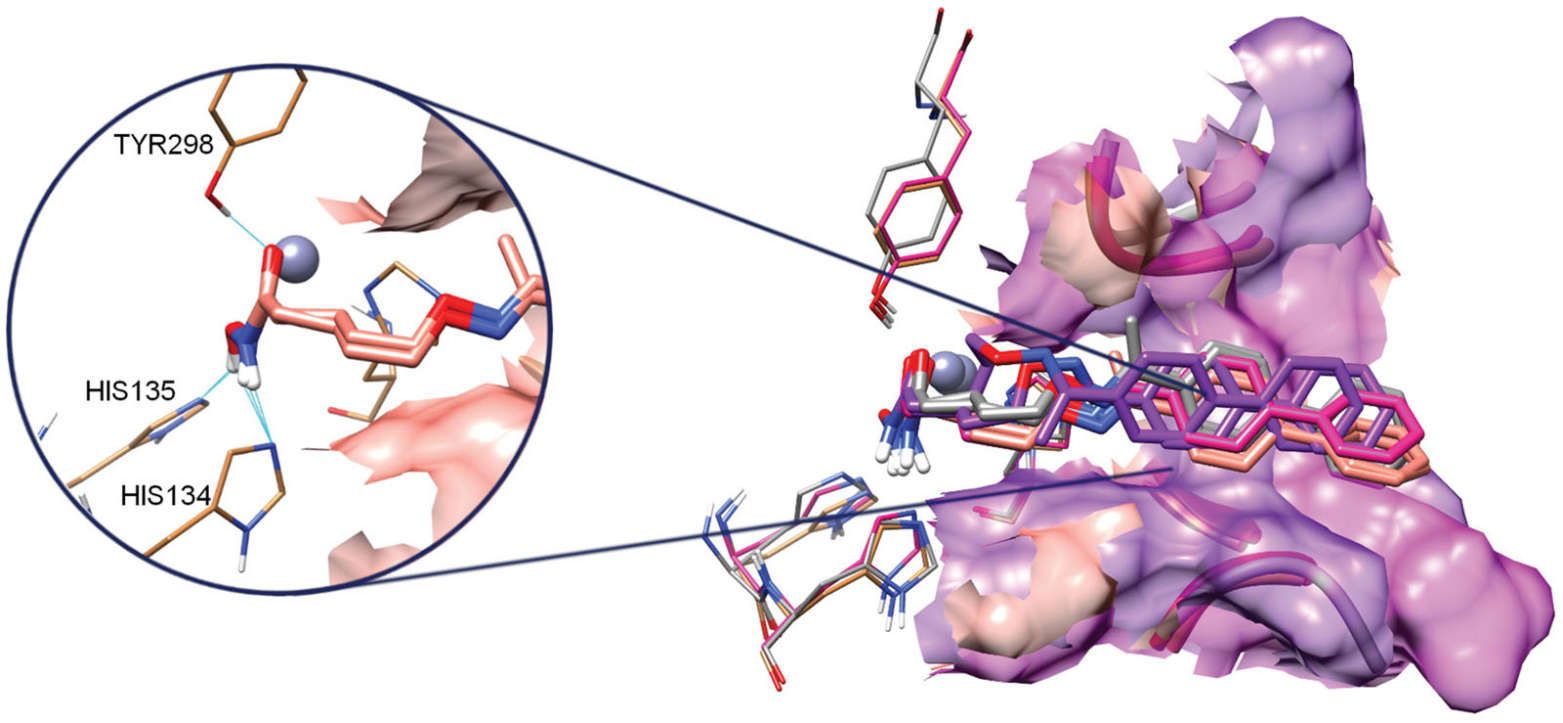
Docking poses of inhibitors **SN2** and **VS16**. The colour code is protein dependent: the complex of both inhibitors in HDAC1, HADC3, HDAC6 and HDAC8 are magenta, salmon, grey, and purple coloured, respectively. On the left are highlighted the hydrogen bonds engaged by both compounds with second shell catalytic residues of HDAC3.

Moreover, the computational analysis of compounds **SN2** and **VS16** showed that both ligands are similarly located in the four HDAC binding channel subtypes (Figure 6). The biphenyl CAP of these ligands is longer than the one present in **LD10** and **VS13** and it linearly crossed the outer binding regions between loop3 and 4.

As previously mentioned, the loop3 of HDAC8 shrinked the outer binding channel. This conformation pushed the CAP of **SN2** and **VS16** inhibitors deeply (more than 2 Å) towards the catalytic core. This scrunched conformation caused a detrimental folding of the spacer and allowed only one polar interaction with second catalytic shell residues.

In the other HDAC subtypes, all **SN2** and **VS16** poses were quite superposed. In the outer portion of the binding site, the biphenyl CAP was located close to loop3 (in a ranking HDAC3 > HDAC1 > HDAC6) while, in the inner binding pocket, the number of hydrogen bonds increased from one in HDAC8 to two in HDAC6 and HDAC1, and finally three in HDAC3 (not shown for clarity in Figure 6).

For all investigated ligands (**LD10**, **VS13**, **SN2** and **VS16**) the computational study showed tiny differences in the ligand-binding poses, principally due to the sequence and shape variations of loops3 and 4 among the four HDAC subtypes.

However, the slight differences of the ligand-binding mode are crucial to strengthen or decrease the inhibitor interactions and consequently binding affinity^30^.

### 2.4. Activity on human UM cell lines in vitro

**Figure 7.**
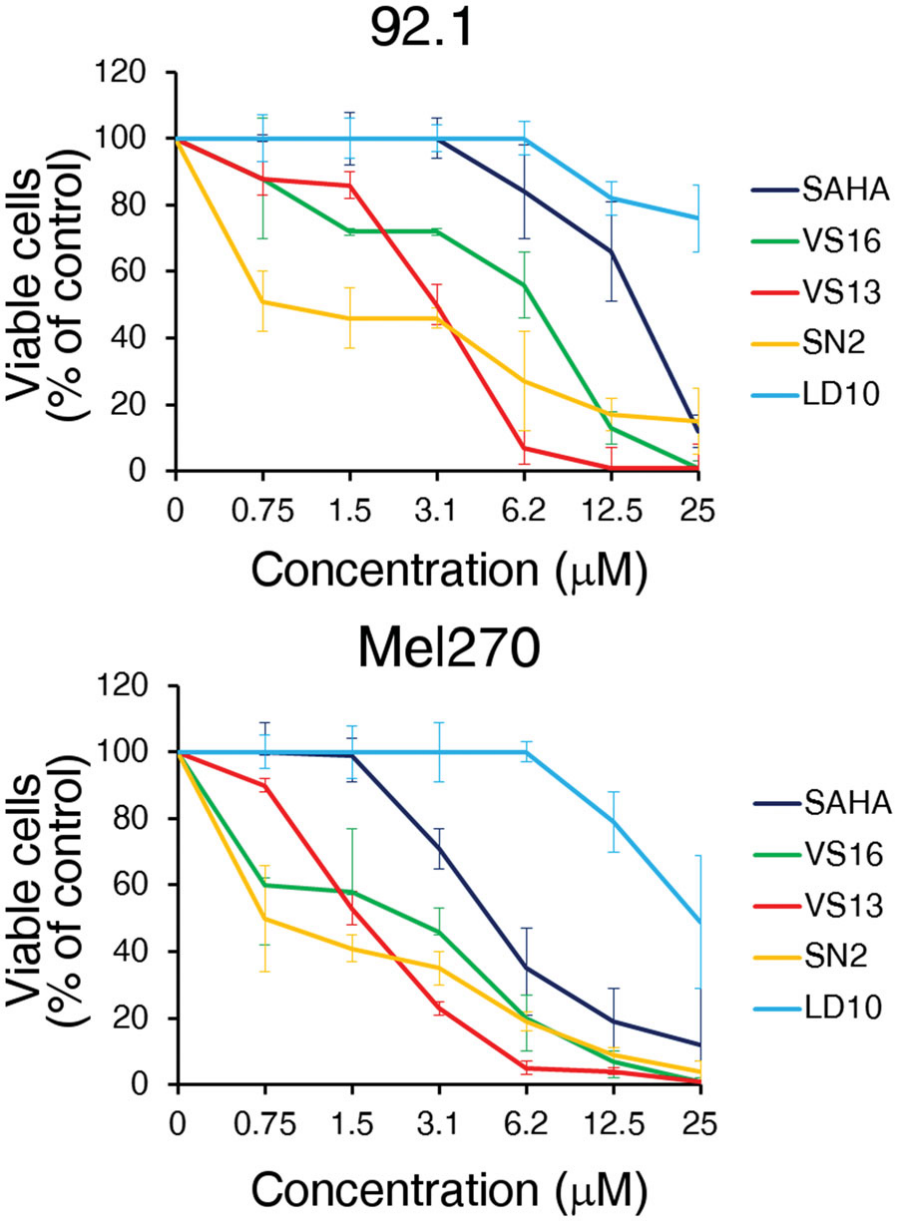
MTT. *In vitro* cytotoxicity at the 72 h time point of the different compounds in human UM cell lines 92.1 and Mel270, as assessed by the MTT cell viability assay. Data are expressed as percent of control with the DMSO solvent, which was used at the same amount present in the highest compound concentration. Error bars represent SD of quadruplicates. One representative experiment is shown.

The anti-proliferative effects of the most promising HDAC inhibitors, **VS16**, **VS13**, **SN2**, and **LD10** were evaluated on human UM cell lines (92.1 and Mel270) and SAHA was used as a positive control. First, we assessed by MTT assay the cell viability after a 72 h treatment at different concentrations. Figure 7 and Supplementary Figure S2 show that compounds **VS16**, **VS13**, and **SN2** have a stronger effect on 92.1 and Mel270 cell viability than SAHA and **LD10**. Similar results were found for ovarian carcinoma cell line A2780 and to a lesser extent for SKOV3 (Supplementary Figure S2).

**Figure 8.**
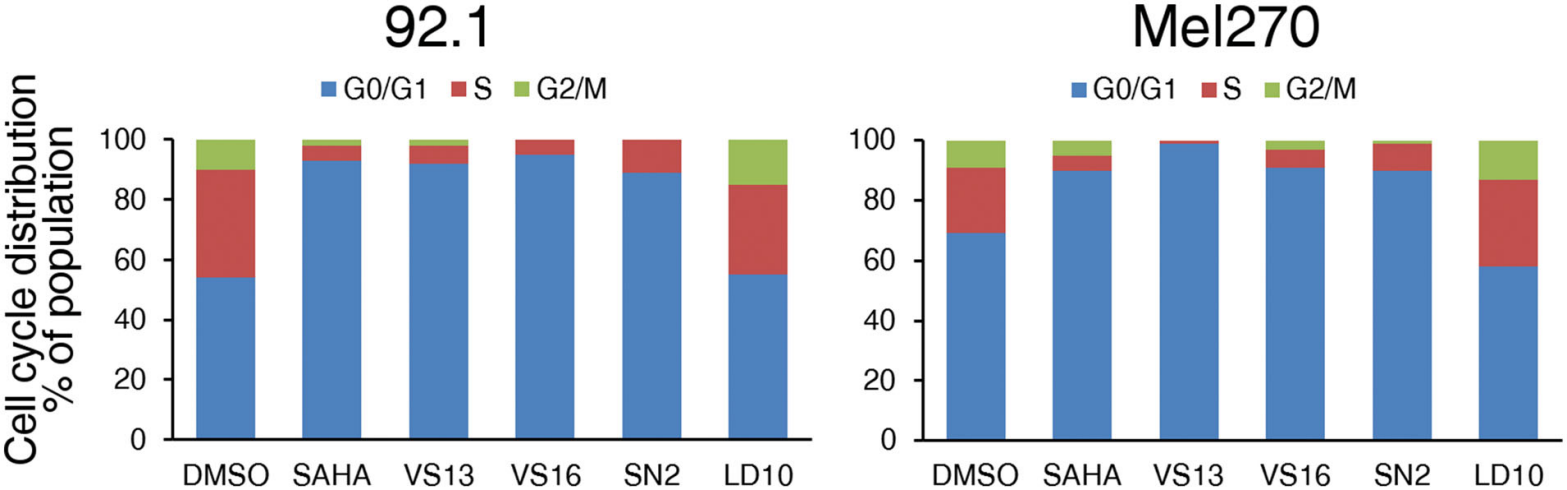
Influence of the different compounds and DMSO solvent control on cell cycle distribution in UM 92.1 and Mel270 cells. The cells were treated with 10 µM compound for 48 h and cell cycle distribution was analysed by flow cytometry after fixation and staining with PI. The percentage of cells in each category is indicated. One representative experiment is shown.

We then investigated whether this anti-proliferative effect was due to the blockade in the G0/G1 phase of the cell cycle, as expected for SAHA^31^. Flow cytometry analysis of cell cycle distribution in 92.1 and Mel270 cells after treatment with the different compounds or control DMSO, indeed showed that **VS16, VS13**, **SN2**, and SAHA efficiently block the cell cycle at the G0/G1 phase (Figure 8 and Supplementary Figure S3) while **LD10** behaved like the control DMSO.

**Figure 9.**
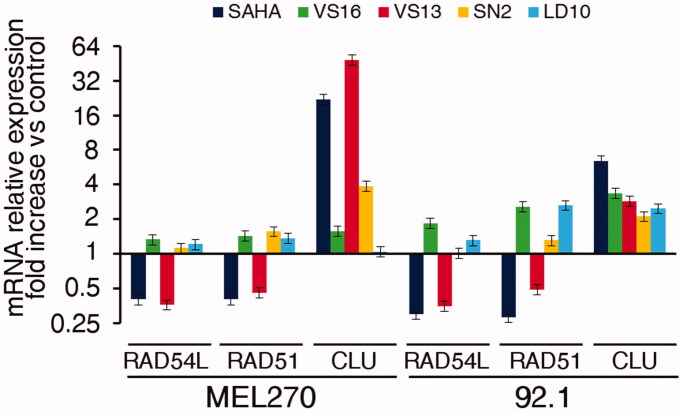
Modulation of RAD54L, RAD51 and CLU mRNA expression by the different compounds in UM cells 92.1 and Mel270. Cells were treated for 48 h with 10 µM compound or the corresponding amount of DMSO. Data, normalised to GAPDH housekeeping gene, are expressed as fold change relative to the DMSO control. Error bars represent SD of triplicates. One representative experiment is shown.

Finally, we determined if the treatment of UM cells 92.1 and Mel270 with our compounds could modify the mRNA levels of HDAC target genes. We chose genes both down-regulated (*RAD54L*, *RAD51*) and up-regulated (*CLU*, *DHRS2*, and *CDKN1A*) by HDAC inhibitors in cancer cells^32^^,^^33^. As shown in Figure 9 and Supplementary Figure S4, **VS13** induced a pattern of gene modulation, which overlapped that of SAHA. Of note, **VS13** shares with SAHA the high activity on HDAC6 (see Table 2), thus supporting the hypothesis that these modulatory effects on genes could be mainly mediated by HDAC6 inhibition on which the other compounds have a lower effect.

## 3. Conclusions

UM represents an aggressive type of cancer and currently, there is no effective treatment for metastatic UM. This cancer form is characterised by an overexpression of HDACs and HDAC inhibitors have been shown to inhibit the growth of UM *in vivo* and *in vitro*. In this study, we synthesised a series of HDACIs based on SAHA scaffold bearing an (arylidene)aminoxy moiety. All new compounds were tested first on HeLa nuclear extract to evaluate if the chemical modification of the SAHA structure maintained the HDAC inhibitory activity. Among all the synthesised hydroxamates we tested the most active ones on isolated enzymes, HDAC1, 3, 6, and 8. The best inhibitory results were obtained with compound **VS13**, bearing a quinoline as the CAP group, which showed a nanomolar affinity for HDAC6 (IC_50_=10 nM) and a 1000-fold selectivity over HDAC8. On these four hydroxamic acid derivatives docking studies were performed to explain their binding mode and the interaction inside the HDAC1, 3, 6, and 8 active sites. In particular, the nitrogen of the quinoline ring was shown to play an important role by forming a strong hydrogen bond with the backbone of loop3 present in HDAC6 catalytic site. The effect of HDAC inhibition in living cells was evaluated by testing the most promising compounds on human UM cell lines, 92.1 and Mel270, in comparison with SAHA. **VS13** showed a strong antiproliferative activity by MTT assay and the ability to efficiently block the cell cycle at the G0/G1 phase, similarly to SAHA. Finally, we determined if treatment with these compounds could modify the mRNA levels of HDAC target genes in UM cells and **VS13** induced a pattern of gene modulation which overlapped that of SAHA. Considering the particular activity of **VS13** on HDAC6, these results support the hypothesis that the modulatory effects on genes could be mainly mediated by HDAC6 inhibition. However, additional studies will be necessary to shed light on the mechanism involved in the antitumor activity of HDACIs in this aggressive type of cancer.

## 4. Experimental section

### 4.1. Chemistry

Analytical grade reagents and solvents were purchased from Sigma-Aldrich (St. Louis, MO), and were used as supplied. Melting points were determined on a Köfler hot-stage apparatus and are uncorrected. ^1^H and ^13^C NMR spectra were recorded on a Bruker Avance III HD 400 MHz spectrometer (Fällander, Switzerland) and on a Varian Gemini-200 MHz spectrometer. Chemical shifts (*δ*) are reported in parts per million and coupling constants (*J*) are reported in hertz (Hz). ^13^C NMR spectra were fully decoupled. The following abbreviations were used to explain multiplicities: singlet (s), doublet (d), triplet (t), double doublet (dd), broad (br), and multiplet (m). Chromatographic separations were performed on silica gel columns by flash column chromatography (Kieselgel 40, 0.040–0.063 mm, Merck). Reactions were monitored by thin-layer chromatography (TLC) on silica gel plates containing a fluorescent indicator (Merck Silica Gel 60 F254) and spots were detected under UV light (254 nm) and hydroxamic acids were visualised with FeCl_3_ aqueous solution. Evaporation was carried *in vacuo* (rotating evaporator). Sodium sulphate was always used as the drying agent. Commercially available chemicals were purchased from Sigma-Aldrich. The oxime **5d** is commercially available and was purchased from Sigma-Aldrich. Elemental analysis was used to determine the purity of target compounds and was performed by our analytical laboratory and agreed with the theoretical values to within ±0.4%.

### 4.2. General procedure for the synthesis of oximes 5a–c, e, f

A solution of the appropriate commercially available aldehyde or ketone (3.21 mmol) and NH_2_OH^.^HCl (6 mmol) in a mixture of EtOH 98% (5.3 ml) and Pyridine (8.3 mmol) was stirred at 150 °C for 150 min. Then, the solvent was evaporated and the crude was added with a saturated solution of NaHCO_3_, filtered and washed with water to obtain the oximes **5a–c,e,f**.

#### 4.2.1. (E)-4-(methylthio)benzaldehyde oxime (5a)

The title compound was prepared from the appropriate aldehyde following the general procedure to obtain **5a** as a white solid (64% yield). ^1^HNMR (CDCl_3_):8.03 (s, 1H); 7.52–7.48 and 7.25–7.21 (m, 4H, *J* = 8.42 Hz), 2.51 (s, 3H).

#### 4.2.2. (E)-1,1′-biphenyl-4-carbaldehyde oxime (5b)

The title compound was prepared from the appropriate aldehyde following the general procedure to obtain **5b** as a white solid (99% yield). ^1^H NMR (200 MHz, CDCl_3_) *δ*: 8.19 (s, 1H); 7.88–7.83 and 7.55–7.47 (m, 8H);

#### 4.2.3. (E)-2-naphthaldehyde oxime (5c)

The title compound was prepared from the appropriate aldehyde following the general procedure to obtain **5c** as a white solid (64% yield). ^1^H NMR (200 MHz, CDCl_3_) *δ*: 8.30 (s, 1H); 7.88–7.83 and 7.55–7.47 (m, 8H).

#### 4.2.4. 1-([1,1′-Biphenyl]-4-yl)ethanone oxime(5e)

The title compound was prepared from the appropriate ketone following the general procedure to obtain **5e** as a white solid (96% yield). ^1^H NMR (200 MHz, CDCl_3_) *δ*: 7.74–7.59 (m, 6H); 7.49–7.34 (m, 3H); 2,33 (s, 3H).

#### 4.2.5. 1-([1,1′-Biphenyl]-4-yl)ethanone oxime(5f)

The title compound was prepared from the appropriate aldehyde following the general procedure to obtain **5f** as a white solid (90% yield). ^1^H NMR (200 MHz, CDCl_3_) *δ*: 8.43 (s, 1H), 8.18–8.09 (m, 3H), 7.97–7.93 (m, 1H), 7.84–7.70 (m, 1H), 7.60–7.52 (m,1H).

### 4.3. General procedure for the synthesis of ethyl (E)–3–((arylideneamino)oxy)propanoates 6a–c

To a solution of the appropriate oxime **5a–c** (3.36 mmol) in MeCN (1.5 ml), triphenylphosphine (0.67 mmol) and ethyl acrylate (3.36 mmol) were added. The obtained solution was stirred at 65 °C for 72 h. The reaction mixture was dried under reduced pressure to give a crude oil that was purified by flash chromatography on silica gel. The evaporation of the selected fractions gave a pure yellow oil constituted exclusively by the *E* isomer.

#### 4.3.1. Ethyl (E)-3-(((4-(methylthio)benzylidene)amino)oxy)propanoate (6a)

The title compound was prepared from the oxime **5a** following the general procedure. The crude product was purified by flash chromatography on silica gel (n-hexane/EtOAc 7:3) to give a yellow oil (30% yield). ^1^H NMR (200 MHz, CDCl_3_) *δ*: 8.03 (s, 1H), 7.51–7.47 (m, 2H), 7.28–7.21 (m, 2H), 4.44 (t, *J* = 6.41 Hz, 2H), 4.19 (q, *J* = 7.14 Hz, 2H), 2.75 (t, *J* = 6.41 Hz, 2H), 2.51 (s, 3H), 1.28 (t, *J* = 7.14 Hz, 3H).

#### 4.3.2. Ethyl (E)-3-((([1,1′-biphenyl]-4-ylmethylene)amino)oxy)propanoate (6b)

The title compound was prepared from the oxime **5b** following the general procedure. The crude product was purified by flash chromatography on silica gel (n-hexane/EtOAc 7:3) to give a yellow oil (35% yield). ^1^H NMR (200 MHz, CDCl_3_) *δ*: 8.11 (s, 1H), 7.49–7.39 (m, 3H), 7.64–7.57 (m, 6H), 4.46 (t, *J* = 6.41 Hz, 2H), 4.19 (q, *J* = 7.14 Hz, 2H), 2.76 (t, *J* = 6.41 Hz, 2H), 1.27 (t, *J* = 7.14 Hz, 3H).

#### 4.3.3. Ethyl (E)-3-(((naphthalen-2-ylmethylene)amino)oxy)propanoate (6c)

The title compound was prepared from the oxime **5b** following the general procedure. The crude product was purified by flash chromatography on silica gel (n-hexane/EtOAc 9:1) to give a yellow oil (42% yield). ^1^H NMR (200 MHz, CDCl_3_) *δ*: 8.21 (s, 1H), 7.84–7.82 (m, 5H), 7.52–7.47 (m, 2H), 4.49 (t, *J* = 6.41 Hz, 2H), 4.18 (q, *J* = 7.14 Hz, 2H), 2.77 (t, *J* = 6.41 Hz, 2H), 1.27 (t, *J* = 7.14 Hz, 3H),

### 4.4. General procedure for the synthesis of ethyl (E)–4–((arylideneamino)oxy)pentanoates (7a–f)

To a cooled (0 °C) and stirred solution of the appropriate oxime **5a–f** (4.69 mmol) and ethyl 5-bromopentanoate (3.91 mmol) in anhydrous DMF (25 ml), Cs_2_CO_3_ (4.69 mmol) was added. The resulting mixture was stirred for 24 h at room temperature. Then, water was added and the solution was extracted with Et_2_O. The organic phases were combined, dried with Na_2_SO_4_, filtered, and evaporated under vacuum. The crude was purified affording exclusively the *E* isomer.

#### 4.4.1. Ethyl (E)-5-(((4-(methylthio)benzylidene)amino)oxy)pentanoate (7a)

The title compound was prepared from the oxime **5a** following the general procedure. The crude product was purified by flash chromatography on silica gel (n-hexane/EtOAc 6:4) to give a yellow oil (48% yield). ^1^H NMR (200 MHz, CDCl_3_) *δ*: 8.03 (s, 1H), 7.23 (m, 2H), 7.50 (m, 2H), 4.09–4.21 (m, 4H), 2.51 (s, 3H), 2.41–2.32 (m, 2H), 1.77–1.80 (m, 4H),1.26 (t, *J* = 7.14 Hz, 3H).

#### 4.4.2. Ethyl (E)-5-((([1,1′-biphenyl]-4-ylmethylene)amino)oxy)pentanoate (7b)

The title compound was prepared from the oxime **5b** following the general procedure. The crude product was purified by flash chromatography on silica gel (n-hexane/EtOAc 7:3) to give a yellow oil (85% yield). ^1^H NMR (200 MHz, CDCl_3_) *δ*: 8.01 (s, 1H), 7.74–7.59(m, 6H), 7.48–7.32 (m, 3H) 4.25–4.17 (m, 2H), 4.21 (q, *J* = 7.14 Hz, 2H), 2.37–2.34 (m, 2H), 1.80–1.74 (m, 4H), 1.26 (t, *J* = 7.14 Hz, 3H).

#### 4.4.3. Ethyl (E)-5-(((naphthalen-2-ylmethylene)amino)oxy)pentanoate (7c)

The title compound was prepared from the oxime **5c** following the general procedure. The crude product was purified by flash chromatography on silica gel (n-hexane/EtOAc 6:4) to give a yellow oil (65% yield). ^1^H NMR (200 MHz, CDCl_3_) *δ*: 8.22 (s, 1H), 7.85–7.78 (m, 5H), 7.53–7.47 (m, 2H), 4.25–4.20 (m, 2H), 4.18–4.08 (q, *J* = 7.14 Hz, 2H), 2.41–2.35 (m, 2H), 1.82–1.75 (m, 4H), 1.25 (t, *J* = 7.14 Hz, 3H).

#### 4.4.4. Ethyl (E)-4-((benzylideneamino)oxypentanoate (7d)

The title compound was prepared from the oxime **5d** following the general procedure. The crude product was purified by flash chromatography on silica gel (n-hexane/EtOAc 6:4) to give a yellow oil (80% yield). ^1^H NMR (200 MHz, CDCl_3_) 8.07 (s,1H), 7.59–7.55(m, 2H), 7.37–7.35 (m, 3H), 4.21–4.14 (m, 2H), 4.12 (q, *J* = 7.14 Hz, 2H), 2.40–2.33 (m, 2H), 1.79–1.72 (m, 4H), 1.25 (t, *J* = 7.14 Hz, 3H),

#### 4.4.5. Ethyl (E)-4-(((1-([1,1′-biphenyl]-4-yl)ethylidene)amino)oxy)butanoate (7e)

The title compound was prepared from the oxime **5e** following the general procedure. The crude product was purified by flash chromatography on silica gel (n-hexane/EtOAc 6:4) to give a yellow oil (66% yield). ^1^H NMR (200 MHz, CDCl_3_) *δ*: 8.02 (s, 1H), 7.74–7.59 (m, 6H), 7.48–7.32 (m, 3H), 4.25–4.20 (m, 2H), 4.21 (q, *J* = 7.14 Hz, 2H), 2.41–2.30 (m, 2H), 2.26 (s, 3H), 1.94–1.78 (m, 4H), 1.26 (t, *J* = 7.14 Hz, 3H).

#### 4.4.6. Ethyl (E)-4-(((quinolin-3-ylmethylene)amino)oxy)butanoate (7f)

The title compound was prepared from the oxime **5e** following the general procedure. The crude product was purified by flash chromatography on silica gel (n-hexane/EtOAc 7:3) to give a yellow oil (90% yield). ^1^H NMR (200 MHz, CDCl_3_) *δ*: 8.30 (s, 1H), 8.14–7.94 (m,3H), 7.82–7.66 (m, 2H), 7.57–7.49 (m, 1H), 4.27 (m, 2H), 4.12 (q, *J* = 7.14 Hz, 2H), 2.40–2.32 (m, 2H), 1.80–1.72 (m, 4H), 1.24 (t, *J* = 7.14 Hz, 3H).

### 4.5. General procedure for the synthesis of (E)–N–hydroxy–3–((arylideneamino)oxy)propanamides (1a–c)

To a stirred solution of KOH (1.32 mmol) in MeOH (2 ml) was added a 50% aqueous solution of NH_2_OH (0.089 ml). This solution was added dropwise in 30 min to a stirred and cooled solution (0 °C) of the appropriate ethyl ester **6a−c** (0.087 mmol) in MeOH (2 ml).

After stirring at 0 °C for 30 min. and at room temperature overnight the mixture was evaporated *in vacuo***.** The residue was added with water and the aqueous solution washed with Et_2_O. The aqueous phase was acidified with 10% HCl to pH = 5 and then extracted with EtOAc. Organic layers were collected, dried, and evaporated *in vacuo* to give a solid.

#### 4.5.1. (E)-N-hydroxy-3-(((4-(methylthio)benzylidene)amino)oxy)propanamide(1a)

The title compound was prepared from the ethyl ester **6a** following the general procedure. The crude product was purified by flash chromatography on silica gel (n-hexane/EtOAc 3:7) to give a semisolid (40% yield). ^1^H NMR (400 MHz, DMSO-d_6_): *δ* 10.48 (bs, 1H), 8.81 (bs, 1H), 8.11 (s, 1H), 7.55–7.53 (m, 2H), 7.33–7.28 (m, 2H); 4.35 (t, *J* = 6 Hz, 2H), 2.50 (s, 3H), 2.41 (t, *J* = 6 Hz, 2H). ^13^C (100 MHz, DMSO-d_6_): *δ* 167.1, 148.5, 141.0, 128.9, 128.4, 127.7, 126.2, 70.3, 32.9, 14.8. Elemental analysis calcd. (%) for C_11_H_14_N_2_O_3_S: C 51.95; H 5.55; N 11.02; found: C 52.02, H 5.61, N 11.25.

#### 4.5.2. (E)-3-((([1,1′-biphenyl]-4-ylmethylene)amino)oxy)-N-hydroxypropanamide (1b)

The title compound was prepared from the ethyl ester **6b** following the general procedure. The crude product was purified by flash chromatography on silica gel (n-hexane/EtOAc 3:7) to give a yellow solid. (40% yield). M.p. = 128–130 °C. ^1^H NMR (400 MHz, DMSO-d_6_) *δ*: 10.47 (s, 1H), 8.79 (s, 1H), 8.26 (s, 1H),7.76–7.69 (m, 6H, Ar), 7.57–7.46 (m, 2H, Ar), 7.41–7.38 (m, 1H, Ar), 4.32 (t, *J =* 6.4 Hz, 2H), 2.39 (t, *J =* 6.4 Hz, 2H). ^13^C NMR (100 MHz, DMSO-d_6_): *δ* 167.0, 149.1, 141.9, 139.8, 131.9, 129.5, 128.3, 127.9, 127.5, 127.1, 70.3, 33.0. Elemental analysis calcd. (%) for C_16_H_16_N_2_O_3:_ C, 67.59; H, 5.67; N, 9.85; found: C, 69.99, H, 6.02, N, 10.02.

#### 4.5.3. (E)-N-hydroxy-3-(((naphthalen-2-ylmethylene)amino)oxy)propanamide (1c)

The title compound was prepared from the ethyl ester **6c** following the general procedure. The crude product was purified by flash chromatography on silica gel (n-hexane/EtOAc 3:7) to give a yellow solid. (47% yield). M.p. = 153–155 °C; ^1^HNMR (400 MHz, DMSO-d_6_) *δ*: 10.48 (bs, 1H), 8.80 (s,1H), 8.35 (s, 1H), 8.07 (s, 1H), 7.95–7.93 (m, 3H), 7.81 (m, 1H), 7.57–7.55 (m, 2H), 4.35 (t, *J* = 6.24 Hz, 2H), 2.41 (t, *J* = 6.24 Hz, 2H). ^13^C NMR (100 MHz, DMSO-d_6_) *δ*: 167.1, 149.4, 134.0, 133.2, 130.1, 128.9, 128.8, 128.7, 128.2, 127.6, 127.3, 127.1, 70.3, 33.0, 14.9. Elemental analysis calcd. (%) for C_14_H_14_N_2_O_3_: C, 65.11; H, 5.46; N, 10.85; found: C, 65.19, H, 5.52, N, 10.92.

### 4.6. General procedure for the synthesis of (E)–3–((arylideneamino)oxy)pentananamide (2a–f)

To a stirred solution of KOH (2.47 mmol) in MeOH (4 ml) was added a 50% aqueous solution of NH_2_OH (0.49 ml). This solution was added dropwise in 30 min to a stirred and cooled solution (0 °C) of the appropriate ethyl ester **7a−f** (3.12 mmol) in MeOH (2 ml).

After stirring at 0 °C for 30 min. and at room temperature overnight the mixture evaporated *in vacuo***.** The residue was added with water and the aqueous solution washed with Et_2_O. Aqueous phase was acidified with 10% HCl to pH = 5 and then extracted with EtOAc. Organic layers were collected, dried, and evaporated *in vacuo* to give a crude solid.

#### 4.6.1. (E)-N-hydroxy-5-(((4-(methylthio)benzylidene)amino)oxy)pentanamide (2a)

The title compound was prepared from the ethyl ester **7a** following the general procedure. The crude product was purified by flash chromatography on silica gel (n-hexane/EtOAc 3:7) to give a yellow semisolid. (45% yield); ^1^H NMR (400 MHz, DMSO-d_6_*)*: *δ* 10.48 (bs, 1H), 8.81 (bs, 1H), 8.18 (s, 1H), 7.55–7.53 (m, 2H), 7.33–7.28 (m, 2H); 4.09 (t, *J* = 6 Hz, 2H), 2.62 (t, *J* = 6 Hz, 2H), 2.50 (s, 3H), 1.68–1.57 (m, 4H). ^13^C (100 MHz, DMSO-d_6_): *δ* 167.1, 148.5, 141.0, 128.9, 128.4, 127.7, 126.2, 70.3, 32.9, 28.2, 21.6. Elemental analysis calcd. (%) for C_13_H_18_N_2_O_3_S: C, 55.30; H, 6.43; N, 9.92; found: C, 55.39, H, 6.52, N, 10.02.

#### 4.6.2. (E)-5-((([1,1′-biphenyl]-4-ylmethylene)amino)oxy)-N-hydroxypentanamide (2b) (SN2)

The title compound was prepared from the ethyl ester **7b** following the general procedure. The crude product was purified by flash chromatography on silica gel (n-hexane/EtOAc 3:7) to give a yellow solid (35% yield). M.p. =160–162 °C. ^1^HNMR (400 MHz, DMSO-d_6_): *δ* 8.27 (s, 1H),7.73–7.69 (m, 6H, Ar), 7.50–7.46 (m, 2H, Ar), 7.40–7.38 (m, 1H, Ar), 4.10 (m, 2H), 1.84 (t, *J =* 7.1 Hz, 2H), 1.64–1.47 (m, 4H). ^13^C NMR (100 MHz, DMSO-d_6_): *δ* 175.5, 148.27, 141.7, 139.8, 131.8, 129.4, 128.3, 127.9, 127.5, 127.1, 74.5, 29.6, 23.5. Elemental analysis calcd. (%) for C_18_H_20_N_2_O_3_: C, 69.21; H, 6.45; N, 8.97; found: C, 69.33, H, 6.52, N, 9.02.

#### 4.6.3. (E)-N-hydroxy-5-(((naphthalen-2-ylmethylene)amino)oxy)pentanamide (2c) (LD10)

The title compound was prepared from the ethyl ester **7c** following the general procedure. The crude product was purified by flash chromatography on silica gel (n-hexane/EtOAc 3:7) to give a yellow solid. (35% yield). M.p. = 229–231 °C. ^1^HNMR (400 MHz, DMSO-d_6_) *δ*: 10.53 (bs, 1H), 8.81 (bs, 1H), 8.37 (s, 1H), 8.06 (s, 1H), 7.95–7.92 (m, 3H), 7.83–7.81 (m, 1H), 7.57–7.54 (m, 2H), 4.13 (t, *J* = 6.8 Hz, 2H), 1.88 (t, *J* = 7.2 Hz, 2H), 1.67–1.50 (m, 4H). ^13^C NMR (100 MHz, DMSO-d_6_) *δ*: 175.6, 148.9, 134.0, 133.3, 130.4, 128.9, 128.7, 128.5, 128.2, 127.5, 127.2, 123.1, 74.5, 38.9, 29.6, 23.4. Elemental analysis calcd. (%) for *C_16_H_18_N_2_O_3_*: C, 67.12; H, 6.34; N, 9.78; found: C, 67.33, H, 6.42, N, 9.82.

#### 4.6.4. (E)-5-((benzylideneamino)oxy)-N-hydroxypentanamide (2d)

The title compound was prepared from the ethyl ester **7d** following the general procedure. The crude product was purified by flash chromatography on silica gel (n-hexane/EtOAc 3:7) to give a yellow solid. (35% yield). M.p. = 134–136 °C. ^1^HNMR (400 MHz, DMSO-d_6_): *δ* 10.51 (bs, 1H), 8.81 (bs, 1H), 8.22–8.21 (m, 1H, Ar), 7.61–7.59 (m, 2H, Ar), 7.41–7.39 (m, 2H, Ar), 4.11–4.05 (m, 2H), 1.65–1.46 (m, 4H). ^13^C NMR (100 MHz, DMSO-d_6_): *δ* 172.2, 148.8, 132.6, 130.2, 129.2, 127.2, 73.7, 35.6, 28.7, 23.3. Elemental analysis calcd. (%) for C_12_H_16_N_2_O_3_: C, 61.00; H, 6.83; N, 11.86; found: C, 61.13, H, 6.92, N, 11.92.

#### 4.6.5. (E)-5-(((1-([1,1′-biphenyl]-4-yl)ethylidene)amino)oxy)-N-hydroxypentanamide (2e) (VS16)

The title compound was prepared from the ethyl ester **7e** following the general procedure. The crude product was purified by trituration with Et_2_O to give a white solid (35% yield). M.p. = 142 °C; ^1^HNMR (400 MHz, DMSO-d_6_) *δ*: 10.72 (s, 1H), 9.39 (bs, 1H),7.76–7.68 (m, 6H, Ar), 7.50–7.46 (m, 2H, Ar), 7.40–7.38 (m, 1H, Ar), 4.13 (t, *J* = 6.0 Hz, 2H), 3.40–3.28 (m, 2H), 2.12 (s, 3H), 1.90 (t, *J* = 7.1 Hz, 2H), 1.64–1.55 (m, 4H). ^13^C NMR (100 MHz, DMSO-d_6_): *δ* 168.4, 153.7, 141.1, 139.9, 135.6, 129.4, 128.2, 127.1, 126.8, 73.9, 33.2, 29.1, 22.8, 12.7. Elemental analysis calcd. (%) for C_19_H_22_N_2_O_3_: C, 69.92; H, 6.79; N, 8.58; found: C, 69.93, H, 6.82, N, 8.63.

#### 4.6.6. (E)-N-hydroxy-5-(((quinolin-3-ylmethylene)amino)oxy)pentanamide (2f) (VS13)

The title compound was prepared from the ethyl ester **7f** following the general procedure. The crude product was purified by crystallisation with CHCl_3_ to give a white solid (42% yield). M.p. = 113 °C; ^1^HNMR (400 MHz, DMSO-d_6_) *δ*: 10.37 (bs, 1H), 8.68 (s,1H), 8.39 (m, 1H) 8.31 (s, 1H), 8.02 (m, 2H), 7.93 (m, 1H), 7.80 (m, 1H), 7.65 (m, 1H), 4.22 (t, *J* = 6.28 Hz, 2H), 2.01 (t, *J* = 7.2 Hz, 2H), 1.71–1.61 (m, 4H). ^13^C NMR (100 MHz, DMSO-d_6_): *δ* 169.3, 152.1, 149.9, 147.8, 137.3, 130.6, 129.3, 128.5, 128.3, 127.8, 118.1, 74.5, 32.4, 28.6, 22.1. Elemental analysis calcd. (%) for C_15_H_17_N_3_O_3_: C, 62.71; H, 5.96; N, 14.63; found: C, 62.73, H, 6.02, N, 14.69.

### 4.7. General procedure for the synthesis of (E)–3–((arylideneamino)oxy)propanoic acids (3a–c) and (E)–3–((arylideneamino)oxy)pentanoic acids (4a–e)

A solution of the appropriate ethyl ester **6a–c**, **7a–d** (1.35 mmol) in anhydrous EtOH (0.21 ml) was added with a solution of 2 N KOH in anhydrous EtOH (4.90 ml). The resulting mixture was stirred at 40 °C for 24 h. Ethanol was evaporated and the solid residue was dissolved in water and washed with Et_2_O. Aqueous basic phase was then acidified with 1 N HCl until pH = 5 was obtained, and the product was extracted with EtOAc. Organic layers were collected, dried, evaporated to dryness.

#### 4.7.1. (E)-3-(((4-(methylthio)benzylidene)amino)oxy)propanoic acid (3a)

The title compound was prepared from the ethyl ester **6a** following the general procedure white solid (45% yield). M.p. = 76–78 °C. ^1^HNMR (400 MHz, DMSO-d_6_): *δ* 12.28 (bs, 1H), 8.17 (s, 1H), 7.53 (m, 2H, Ar), 7.28 (m, 2H, Ar), 4.28 (t, *J =* 6.24 Hz, 2H), 2.60 (t, *J =* 6.24 Hz, 2H), 2.51 (s, 3H).^13^C NMR (100 MHz, DMSO-d_6_): *δ* 172.8, 149.0, 141.2, 130.1, 128.7, 127.7, 126.1, 125.3, 70.0, 34.7, 14.7. Elemental analysis calcd. (%) for C_11_H_13_NO_3_S: C, 55.21; H, 5.48; N, 5.85; found: C, 55.23, H, 5.52, N, 5.89.

#### 4.7.2. (E)-3-((([1,1′-biphenyl]-4-ylmethylene)amino)oxy)propanoic acid (3b)

The title compound was prepared from the ethyl ester **6b** following the general procedure white solid (48% yield). M.p. = 104–106 °C. ^1^HNMR (400 MHz, DMSO-d_6_): *δ* 8.28 (s, 1H), 7.74–7.69 (m, 6H, Ar), 7.50–7.46 (m, 2H, Ar), 7.41–7.38 (m, 1H, Ar), 4.32 (t, *J =* 6.4 Hz, 2H), 2.63 (t, *J =* 7.1 Hz, 2H), ^13^C NMR (100 MHz, DMSO-d_6_): *δ* 172.8, 149.1, 142.0, 139.8, 131.5, 129.5, 128.3, 127.9, 127.5, 127.13, 70.1, 34.7. Elemental analysis calcd. (%) for C_16_H_15_NO_3_: C, 71.36; H, 5.61; N, 5.20; found: C, 71.43, H, 5.62, N, 5.29.

#### 4.7.3. (E)-3-(((naphthalen-2-ylmethylene)amino)oxy)propanoic acid (3c)

The title compound was prepared from the ethyl ester **6c** following the general procedure white semisolid (56% yield). ^1^HNMR (400 MHz, DMSO-d_6_): *δ* 12.48 (bs, 1H), 8.38 (s, 1H), 8.06 (s, 1H, Ar), 7.96–7.92 (m, 3H, Ar), 7.81 (m, 1H, Ar), 7.57–7.55 (m, 2H, Ar), 4.35 (t, *J =* 6.00 Hz, 2H), 2.66 (t, *J =* 6.20 Hz, 2H).^13^C NMR (100 MHz, DMSO-d_6_): *δ* 173.1, 149.5, 134.0, 133.2, 130.1, 128.9, 128.8, 128.7, 128.2, 127.6, 127.3, 123.1, 70.1, 35.3. Elemental analysis calcd. (%) for C_14_H_13_NO_3_: C, 69.12; H, 5.39; N, 5.76; found: C, 69.13, H, 5.42, N, 5.79.

#### 4.7.4. (E)-5-(((4-(methylthio)benzylidene)amino)oxy)pentanoic acid (4a)

The title compound was prepared from the ethyl ester **7a** following the general procedure white solid (76% yield). M.p. = 70–72 °C; 1HNMR (400 MHz, DMSO-d_6_): *δ* 12.10 (bs, 1H), 8.19 (s, 1H), 7.54 (m, 2H, Ar), 7.31 (m, 2H, Ar), 4.09 (t, *J* = 6.00 Hz, 2H), 2.51 (s, 3H), 2.26 (t, *J* = 7.2 Hz, 2H), 1.68–1.63 (m, 4H). ^13^C NMR (100 MHz, DMSO-d_6_): *δ* 174.8, 148.5, 141.0, 128.9, 128.4, 127.6, 126.1, 73.6, 33.8, 28.6, 21.6, 14.7. Elemental analysis calcd. (%) for C_13_H_17_NO_3_S: C, 58.40; H, 6.41; N, 5.24; found: C, 58.55, H, 6.42, N, 5.29.

#### 4.7.5. (E)-5-((([1,1′-biphenyl]-4-ylmethylene)amino)oxy)pentanoic acid (4b)

The title compound was prepared from the ethyl ester **7b** following the general procedure white solid (64% yield). M.p. = 98–100 °C; ^1^HNMR (400 MHz, DMSO-d_6_): *δ* 12.02 (bs, 1H),8.28 (s, 1H), 7.74–7.68 (m, 6H, Ar), 7.50–7.46 (m, 2H, Ar), 7.40–7.38 (m, 1H, Ar), 4.13 (t, *J =* 6.4 2H), 2.27 (t, *J =* 7.2 Hz, 2H), 1.70–1.58 (m, 4H). ^13^C NMR (100 MHz, DMSO-d_6_): *δ* 174.8, 148.6, 141.8, 139.8, 131.6, 129.5, 128.3, 127.8, 127.5, 127.1, 73.7, 33.8, 28.6, 21.6. Elemental analysis calcd. (%) for C_18_H_19_NO_3_: C, 72.71; H, 6.44; N, 4.71; found: C, 72.73, H, 6.46, N, 4.79.

#### 4.7.6. (E)-5-(((naphthalen-2-ylmethylene)amino)oxy)pentanoic acid (4c)

The title compound was prepared from the ethyl ester **7c** following the general procedure. Orange solid (55% yield). M.p. = 68–70 °C; ^1^HNMR (400 MHz, DMSO-d_6_) *δ*: 12.01 (bs, 1H), 8.38 (s, 1H), 8.06 (s, 1H, Ar), 7.96–7.92 (m, 3H, Ar), 7.81 (m, 1H, Ar), 7.57–7.54 (m, 2H, Ar), 4.16 (t, *J* = 6.4 Hz, 2H), 2.28 (t, *J* = 7.2 Hz, 2H). ^13^C NMR (100 MHz, DMSO-d_6_): *δ* 174.8, 149.0, 134.0, 133.2, 130.2, 128.9, 128.7, 128.6, 128.2, 127.5, 127.2, 123.1, 73.8, 33.9, 28.6, 21.6. Elemental analysis calcd. (%) for C_16_H_17_NO_3_: C, 70.83; H, 6.32; N, 5.16; found: C, 70.93, H, 6.36, N, 5.19.

#### 4.7.7. (E)-5-((benzylideneamino)oxy)pentanoic acid (4d)

The title compound was prepared from the ethyl ester **7d** following the general procedure. Colourless oil (47% yield). ^1^H NMR (400 MHz, DMSO-d_6_): *δ* 12.03 (bs, 1H), 8.23 (s, 1H), 7.61–7.59 (m, 2H), 7.42–7.39 (m, 3H); 4.10 (t, *J* = 6 Hz, 2H), 2.26 (t, *J* = 6 Hz, 2H), 1.68–1.55 (m, 4H). ^13^C (100 MHz, DMSO-d_6_): *δ* 175.3, 149.4, 133.0, 130.7, 129.7, 127.7, 74.1, 34.3, 29.2, 22.0. Elemental analysis calcd. (%) for C_12_H_15_NO_3_: C, 65.14; H, 6.83; N, 6.33; found: C, 65.23, H, 6.86, N, 6.39.

#### 4.7.8. (E)-5-(((1-([1,1′-biphenyl]-4-yl)ethylidene)amino)oxy)pentanoic acid (4e)

The title compound was prepared from the ethyl ester **7e** following the general procedure. White solid (33% yield). M.p. = 137–139 °C; ^1^HNMR (400 MHz, DMSO-d_6_) *δ*: 7.78–7.67 (m, 6H), 7.52–7.37 (m, 3H), 4.11 (t, 2H, *J* = 6.0 Hz), 2.21 (s, 3H), 1.83 (t, 2H, *J* = 7.3 Hz), 1.64–1.60 (m, 2H), 1.52–1.45 (m, 2H). ^13^C NMR (100 MHz, DMSO-d_6_): *δ* 175.5, 153.5, 141.0, 139.9, 135.7, 129.5, 128.2, 127.1, 126.8, 79.6, 74.4, 29.8, 23.6, 12.7. Elemental analysis calcd. (%) for C_19_H_21_NO_3_: C, 73.29; H, 6.80; N, 4.50; found: C, 73.33, H, 6.86, N, 4.59.

### 4.8. HDACs inhibition assays

HDAC inhibitory activity of newly synthesised compounds was evaluated using an HDAC Fluorimetric Assay/Drug Discovery Kit (AK-500, BIOMOL). Briefly, HeLa nuclear extract (0.5 µl/well) was added to each well already containing the assay buffer (control), diluted trichostatin A (standard reference), or various concentrations of HDAC inhibitors and were incubated at 37 °C with 25 µM of Fluor de Lys^TM^ Substrate. Reactions were stopped after 30 min by adding Fluor de Lys^TM^ Developer. After the addition of this developer, the plate was incubated at room temperature for 15 min, the fluorescence intensity of the wells was measured on a fluorometric plate reader with excitation set at 360 nm and emission detection set at 460 nm.

### 4.9. Microfluidic chip–based HDAC inhibition assay

Test compounds were screened against human histone deacetylases (HDACs) 1, 3, 6, and 8 using the Calliper off-chip mobility shift assay technology. All HDACs were purchased from BPS Bioscience and four selective substrates were used according to the targeted isozyme. A (FAM)-labelled Substrate A peptide (purchasable from PerkinElmer, Product Number CLS96000) was synthesised in-house as previously reported^34^ and used for HDAC1 assays. The other three substrates were purchased from PerkinElmer: two (FITC)-labelled peptides (p53 Acetylated Peptide and Histone 4 Acetylated Peptide, Product Number 760512 and 760513 respectively) were used for HDAC 3 and 6 assays, respectively; a (FAM)-labelled peptide (Broad Substrate B, Product Number CLS960007) was employed as a substrate for HDAC8. Test and standard compounds (3-fold serial dilution starting at 30 µM, 10 concentrations) were incubated with purified HDACs and 1 µM of substrate for 60 min at room temperature in an assay buffer composed of 25 mM Tris-HCl (pH 8.0), 137 mM NaCl, 2.7 mM KCl, 1 mM MgCl_2_, and 0.01% BSA. Reactions were performed in duplicate and terminated by the addition of a stop buffer containing 100 mM HEPES, 0.015% Brij-35, 10 mM EDTA, 0.1% CR-3, and 1.5 µM of the pan-HDAC inhibitor Panobinostat. A DMSO concentration of 3.33% characterised both compounds’ titration and controls wells. Buffers components, except for CR-3 (LabChip Coating Reagent 3, purchased from PerkinElmer, Product Number 760050) and Panobinostat (LBH589, purchased from ApexBio Technology)^35^^,^^36^ were purchased from Sigma Aldrich. As standard compounds (positive controls), three well-known HDAC inhibitors were used: Entinostat (MS-275)^37^, SAHA^38^, and Tubastatin A^39^ (all purchased from Selleck Chemicals). In the case of SAHA an additional 15-point dose curve screening was performed over HDACs 3 and 6. Fluorescence intensity of electrophoretically separated substrate and the product was detected using the LabChip EZ Reader and the inhibition percentage values were normalised according to the associated 0% and 100% inhibition control wells. The data were then analysed by non-linear regression using GraphPad Prism 6.01 software^40^ to derive IC_50_ values.

### 4.10. Molecular modelling

Crystal structures of human HDAC1, HDAC3, HDAC6, and HDAC8 (codes: 5ICN^41^, 4A69^42^, 5EDU^43^, and 3F07^44^) were downloaded from Protein Data Bank (PDB) and superposed for aligning their coordinates using Chimaera^45^. Docking of **LD10**, **VS13**, **VS16**, and **SN2** was carried out using the same procedure identified as the best through preliminary cross- and re-docking studies, by means of ASP fitness function of GOLD software^46^. A scaffold match constraint on the hydroxamate position deduced from the crystallographic ligand structures, with a default strength of 5, was applied. The region of interest for docking was defined in GOLD in such a manner that every protein contained all the residues within 10 Å from the superposed co-crystallised ligands. The “allow early termination” command was deactivated. All ligands were submitted to 80 Genetic Algorithm runs, clustering the output orientations using an RMSD cut-off of 1.5 Å. The metal coordination of the zinc ion was set in an octahedral geometry, as in the crystallographic structures. The default GOLD parameters were used for all other settings.

The ligands were built using Maestro^47^ and then subjected, after a minimisation step, to a conformational search of 1,000 steps in a water environment (using the generalized-Born/surface-area model) by means of Macromodel^48^ software. The algorithm used was the Monte Carlo method with the MMFFs force field and a distance-dependent dielectric constant of 1.0. Missing hydrogens were added to the protein according to the predicted protonation state at the physiological pH, 7.0. Asn and Gln residues were absent in the range of interest for the direct contacts of docking; flip corrections were necessary only for the histidines involved in the zinc coordination, which were protonated in such a way to expose the lone pair towards the zinc atom.

### 4.11. Cells and cell treatments

The human UM cell lines 92.1^49^ and Mel270^50^ (kindly provided to RG by M. J. Jager) and the ovarian cancer cell lines SKOV3 (ATTC), A2780 (ICLC) and A2774 (IST Genoa) were grown in RPMI 1640, with 2 mM L-glutamine, 10% heat-inactivated foetal calf serum, and 100 μg/ml penicillin-streptomycin (Lonza) at 37 °C in a 5% CO_2_ incubator. The cells were cultured for no longer than 3 months, when an aliquot of the original stock was thawed, which had been genotyped using the Cell ID^TM^ System (Promega, G9500) and the GeneMapper^®^ software, version 4.0.

Treatments with the compounds were performed with slight differences, according to the final use of the samples. For the cell viability assay with MTT, cells were seeded in 96-well flat-bottom plates in culture medium at 5 × 10^3^ cells/well, whereas for cell cycle analysis and QRT-PCR cells were seeded in 24-well plates at 5 × 10^4^ cells/well. The day after, a culture medium containing the appropriate amount of compounds or their solvent control DMSO was added, and treatments were carried out for the indicated time.

### 4.12. Evaluation of cell viability and cell cycle status

Cell viability after treatment was evaluated by a microculture tetrazolium reduction assay using MTT [3–(4,5-dimethyltiazol-2-yl) 2,5-diphenyltetrazolium bromide; Sigma]. At 72 h of treatment, 20 μl of MTT stock solution (2 mg/ml in PBS) were added to 200 μl cell cultures for an additional 4 h of incubation. MTT-containing culture medium was then removed and precipitated formazan was dissolved in 100 μl of DMSO. Results were read within 15 min in a microplate reader spectrometer at 540 nm (MEDGENIX 400 ATC, Medgenix Diagnostics), and the means of quadruplicates were calculated. Cell survival was expressed as a percentage of control samples.

To assess the cell cycle status of treated samples, after 48 h of incubation the cells were harvested, washed twice with cold PBS, and fixed in 70% ethyl alcohol at −20 °C overnight. The day after, the samples were washed with cold PBS and incubated for 20 min with propidium iodide (PI) staining solution (50 μg/ml PI, 0.05% Triton X-100, 0.2 mg/ml RNase A in PBS) at room temperature. Flow cytometric analysis of DNA content was performed using a FACScan flow cytometer with the CellQuest software (BD Biosciences). Gating on viable cells was performed using physical parameters and 10^4^ gated events were acquired. Cell cycle analysis on the gated PI distribution was performed using Modfit software.

### 4.13. RT–PCR analysis

To assess the modulation of gene expression, cells were harvested at 48 h of treatment and total RNA was isolated using the NucleoSpin RNA kit (Macherey-Nagel) and reverse-transcribed with the SuperScript III Reverse Transcriptase (Invitrogen). The iQTM SYBR^®^ Green Supermix system (Bio-Rad Laboratories) was used for amplification in the Mastercycler^®^ ep realplex4 instrument (Eppendorf International). Expression levels of mRNAs relative to untreated control were calculated by the *ΔΔ*CT method (Table 3).

## Supplementary Material

Supplemental Material

## References

[1] Yang J, Manson DK, Marr BP, Carvajal RD. Treatment of uveal melanoma: where are we now? Ther Adv Med Oncol 2018;10:1–17. 10.1177/1758834018757175 PMC5824910 29497459

[2] Onken MD, Worley LA, Tuscan MD, Harbour JW. An accurate, clinically feasible multi-gene expression assay for predicting metastasis in uveal melanoma. J Mol Diagn 2010;12:461–8. 20413675 10.2353/jmoldx.2010.090220 PMC2893630

[3] Sharma A, Stei MM, Fröhlich H, et al. Genetic and epigenetic insights into uveal melanoma. Clin Genet 2018;93:952–61. 28902406 10.1111/cge.13136

[4] Croce M, Ferrini S, Pfeer U, Gangemi R. Targeted therapy of uveal melanoma: recent failures and new perspectives. Cancers 2019;11:846. 10.3390/cancers11060846 PMC6628160 31216772

[5] Moschos MM, Dettoraki M, Androudi S, et al. The role of histone deacetylase inhibitors in uveal melanoma: current evidence. Anticancer Res 2018;38:3817–24. 29970501 10.21873/anticanres.12665

[6] Faião-Flores F, Emmons MF, Durante MA, et al. HDAC inhibition enhances the in vivo efficacy of MEK inhibitor therapy in uveal melanoma. Clin Cancer Res 2019;25:5686–701. 31227503 10.1158/1078-0432.CCR-18-3382 PMC6744978

[7] Bi G, Jiang G. The molecular mechanism of HDAC inhibitors in anticancer effects. Cell Mol Immunol 2006;3:285–90. 16978537

[8] Portela A, Esteller M. Epigenetic modifications and human disease. Nat Biotecnol 2010;28:1057–68. 10.1038/nbt.1685 20944598

[9] Falkenberg KJ, Johnstone RW. Histone deacetylases and their inhibitors in cancer, neurological diseases and immune disorders. Nat Rev Drug Discov 2014;13:673–91. 25131830 10.1038/nrd4360

[10] Shan W, Jiang Y, Yu H, et al. HDAC2 overexpression correlates with aggressive clinicopathological features and DNA-damage response pathway of breast cancer. Am J Cancer Res 2017;7:1213–26. 28560068 PMC5446485

[11] Kanno K, Kanno S, Nitta H, et al. Overexpression of histone deacetylase 6 contributes to accelerated migration and invasion activity of hepatocellular carcinoma cells. Oncol Rep 2012;28:867–73. 22766642 10.3892/or.2012.1898

[12] Ropero S, Esteller M. The role of histone deacetylases (HDACs) in human cancer. Mol Oncol 2007;1:19–25. 19383284 10.1016/j.molonc.2007.01.001 PMC5543853

[13] Landreville S, Agapova OA, Matatall KA, et al. Histone deacetylase inhibitors induce growth arrest and differentiation in uveal melanoma. Clin Cancer Res 2012;18:408–16. 22038994 10.1158/1078-0432.CCR-11-0946 PMC3261307

[14] Dai W, Zhou J, Jin B, Pan J. Class III-specific HDAC inhibitor Tenovin-6 induces apoptosis, suppresses migration and eliminates cancer stem cells in uveal melanoma. Scientific Reports 2016;6:22622. 26940009 10.1038/srep22622 PMC4778058

[15] Grant S, Easley C, Kirkpatrick P. Vorinostat. Nat Rev Drug Discovery 2007;6:21–2. 17269160 10.1038/nrd2227

[16] Grant C, Rahman F, Piekarz R, et al. Romidepsin: a new therapy for cutaneous T-cell lymphoma and a potential therapy for solid tumors. Expert Rev Anticancer Ther 2010;10:997–1008. 20645688 10.1586/era.10.88 PMC6361116

[17] Poole R. Belinostat: first global approval. Drugs 2014;74:1543–4. 25134672 10.1007/s40265-014-0275-8

[18] Moore D. Panobinostat (Farydak): a novel option for the treatment of relapsed or relapsed and refractory multiple myeloma. P&T 2016;41:296–300. 27162469 PMC4849337

[19] Ning Z, Li Z, Newman MJ, et al. Chidamide (CS055/HBI-8000): a new histone deacetylase inhibitor of the benzamide class with antitumor activity and the ability to enhance immune cell-mediated tumor cell cytotoxicity. Cancer Chemother Pharmacol 2012;69:901–9. 22080169 10.1007/s00280-011-1766-x

[20] (a) Sangwan R, Rajan R, Mandal PK. HDAC as onco target: reviewing the synthetic approaches with SAR study of their inhibitors. Eur J Med Chem 2018;158:620–706. 30245394 10.1016/j.ejmech.2018.08.073

[21] Roche J, Bertrand P. Inside HDACs with more selective HDAC inhibitors. Eur J Med Chem 2016;121:451–83. 27318122 10.1016/j.ejmech.2016.05.047

[22] Bertrand P. Inside HDAC with HDAC inhibitors. Eur J Med Chem 2010;45:2095. 20223566 10.1016/j.ejmech.2010.02.030

[23] Levinzon L, Madigan M, Nguyen V, et al. Tumour expression of histone deacetylases in uveal melanoma. Ocul Oncol Pathol 2019;5: 153–61. 31049320 10.1159/000490038 PMC6489079

[24] Porter NJ, Osko JD, Diedrich D, et al. Histone deacetylase 6-selective inhibitors and the influence of capping groups on hydroxamate-zinc denticity. J Med Chem 2018;61:8054–60. 30118224 10.1021/acs.jmedchem.8b01013 PMC6136958

[25] Seidel C, Schnekenburger M, Dicato M, Diederich M. Histone deacetylase 6 in health and disease. Epigenomics 2015;7:103–18. 25687470 10.2217/epi.14.69

[26] Wang XX, Wan RZ, Liu ZP. Recent advances in the discovery of potent and selective HDAC6 inhibitors. Eur J Med Chem 2018;143:1406–18. 29133060 10.1016/j.ejmech.2017.10.040

[27] Shen S, Hadley M, Ustinova K, et al. Discovery of a new isoxazole-3-hydroxamate-based histone deacetylase 6 inhibitor SS-208 with antitumor activity in syngeneic melanoma mouse models. J Med Chem 2019;62:8557–77. 31414801 10.1021/acs.jmedchem.9b00946

[28] Berman HM, Westbrook J, Feng Z, et al. The protein data bank. Nucleic Acids Res 2000;28:235–42. 10592235 10.1093/nar/28.1.235 PMC102472

[29] Simossis VA, Heringa J. PRALINE: a multiple sequence alignment toolbox that integrates homology-extended and secondary structure information. Nucleic Acids Res 2005;33:W289–94. 15980472 10.1093/nar/gki390 PMC1160151

[30] Wang D. Computational studies on the histone deacetylases and the design of selective histone deacetylase inhibitors. Curr Top Med Chem 2009;9:241–56. 19355989 10.2174/156802609788085287 PMC2766262

[31] Munster PN, Troso-Sandoval T, Rosen N, et al. The histone deacetylase inhibitor suberoylanilide hydroxamic acid induces differentiation of human breast cancer cells. Cancer Res 2001;61:8492–7. 11731433

[32] Kachhap SK, Rosmus N, Collis SJ, et al. Downregulation of homologous recombination DNA repair genes by HDAC inhibition in prostate cancer is mediated through the E2F1 transcription factor. PLoS One 2010;5:e11208. 20585447 10.1371/journal.pone.0011208 PMC2887841

[33] Glaser KB, Staver MJ, Waring JF, et al. Gene expression profiling of multiple histone deacetylase (HDAC) inhibitors: defining a common gene set produced by HDAC inhibition in T24 and MDA carcinoma cell lines. Mol Cancer Ther 2003;2:151–63. 12589032

[34] Reddy DN, Ballante F, Chuang T, et al. Design and synthesis of simplified largazole analogues as isoform-selective human lysine deacetylase inhibitors. J Med Chem 2016;59:1613–33. 26681404 10.1021/acs.jmedchem.5b01632

[35] Scuto A, Kirschbaum M, Kowolik C, et al. The novel histone deacetylase inhibitor, Lbh589, induces expression of DNA damage response genes and apoptosis in Ph-acute lymphoblastic leukemia cells. Blood 2008;111:5093–100. 18349321 10.1182/blood-2007-10-117762 PMC2384136

[36] Crisanti MC, Wallace AF, Kapoor V, et al. The HDAC inhibitor panobinostat (Lbh589) inhibits mesothelioma and lung cancer cells in vitro and in vivo with particular efficacy for small cell lung cancer. Molecular Cancer Therapeutics 2009;8:2221–31. 19671764 10.1158/1535-7163.MCT-09-0138 PMC3605895

[37] Saito A, Yamashita T, Mariko Y, et al. A synthetic inhibitor of histone deacetylase, Ms-27-275, with marked in vivo antitumor activity against human tumors. Proceedings of the National Academy of Sciences of the United States of America 1999;96:4592–7. 10200307 10.1073/pnas.96.8.4592 PMC16377

[38] Richon VM, Webb Y, Merger R, et al. Second generation hybrid polar compounds are potent inducers of transformed cell differentiation. Proceedings of the National Academy of Sciences of the United States of America 1996;93:5705–8. 8650156 10.1073/pnas.93.12.5705 PMC39124

[39] Butler KV, Kalin J, Brochier C, et al. Rational design and simple chemistry yield a superior, neuroprotective HDAC6 inhibitor, tubastatin A. J Am Chem Soc 2010;132:10842–6. 20614936 10.1021/ja102758v PMC2916045

[40] GraphPad Software 6.01; La Jolla California USA, (2012). www.graphpad.com

[41] Watson PJ, Millard CJ, Riley AM, et al. Insights into the activation mechanism of class I HDAC complexes by inositol phosphates. Nat Commun 2016;7:11262. 27109927 10.1038/ncomms11262 PMC4848466

[42] Watson PJ, Fairall L, Santos GM, Schwabe JWR. Structure of HDAC3 bound to co-repressor and inositol tetraphosphate. Nature 2012;481:335–40. 22230954 10.1038/nature10728 PMC3272448

[43] Hai Y, Christianson DW. Histone deacetylase 6 structure and molecular basis of catalysis and inhibition. Nat Chem Biol 2016;12:741–7. 27454933 10.1038/nchembio.2134 PMC4990478

[44] Dowling DP, Gantt SL, Gattis SG, et al. Structural studies of human histone deacetylase 8 and its site-specific variants complexed with substrate and inhibitors. Biochemistry 2008;47:13554–63. 19053282 10.1021/bi801610c PMC2635894

[45] Pettersen EF, Goddard TD, Huang CC, et al. UCSF chimera-a visualization system for exploratory research and analysis. J Comput Chem 2004;25:1605–12. 15264254 10.1002/jcc.20084

[46] Verdonk ML, Cole JC, Hartshorn MJ, et al. Improved protein-ligand docking using GOLD. Proteins Struct Funct Bioinforma 2003;52:609–23. 10.1002/prot.10465 12910460

[47] Maestro, ver 9.0. Portland (OR): Schrodinger Inc. (1999). Available from: https://www.schrodinger.com/

[48] Macromodel, version 9.7. Portland (OR): Schrödinger Inc. Available from: https://www.schrodinger.com/.

[49] De Waard-Siebinga I, Blom DJ, Griffioen M, et al. Establishment and characterization of an uveal-melanoma cell line. Int J Cancer 1995;62:155–61. 7622289 10.1002/ijc.2910620208

[50] Verbik DJ, Murray TG, Tran JM, Ksander BR. Melanomas that develop within the eye inhibit lymphocyte proliferation. Int J Cancer 1997;73:470–8. 9389558 10.1002/(sici)1097-0215(19971114)73:4<470::aid-ijc3>3.0.co;2-x

