# Supplementary material for: Identification of histone deacetylase inhibitors with (arylidene)aminoxy scaffold active in uveal melanoma cell lines
Source: J Enzyme Inhib Med Chem. 2020 Oct 26;36(1):34–47. doi: 10.1080/14756366.2020.1835883 (PMC7594840; doi:10.1080/14756366.2020.1835883)
Supplement: Supplemental Material [file IENZ_A_1835883_SM9379.pdf]

Supplemental material

Identification of Histone Deacetylase Inhibitors with (Aryliden)aminoxy Scaffold active in Uveal Melanoma Cell Lines

Susanna Nencetti[a,*], Doretta Cuffaro[a], Elisa Nuti[a], Lidia Ciccone[a], Armando Rossello[a,c], Marina Fabbi[d,*], Flavio Ballante[e,f], Gabriella Ortore[a], Grazia Carbotti[d], Francesco Campelli[d], Irene Banti[a], Rosaria Gangemi[d], Garland R. Marshall[e], Elisabetta Orlandini[b,c]

[a]Dipartimento di Farmacia, Universitá di Pisa, Via Bonanno 6, 56126, Pisa, Italy. [b]Dipartimento di Scienze della Terra, Universitá di Pisa, Via Santa Maria 53-55, 56100, Pisa, Italy. [c]Research Center "E. Piaggio", Università di Pisa, Pisa, Italy. [d]IRCCS Ospedale Policlinico San Martino, Genova, Italy. [e]Department of Biochemistry and Molecular Biophysics, Washington University School of Medicine, St. Louis, MO 63110, USA. [f]Present address: Science for Life Laboratory, Department of Cell and Molecular Biology, Uppsala University, BMC Box 596, SE-751 24 Uppsala, Sweden.

Corresponding author:

E-mail address: susanna.nencetti@unipi.it

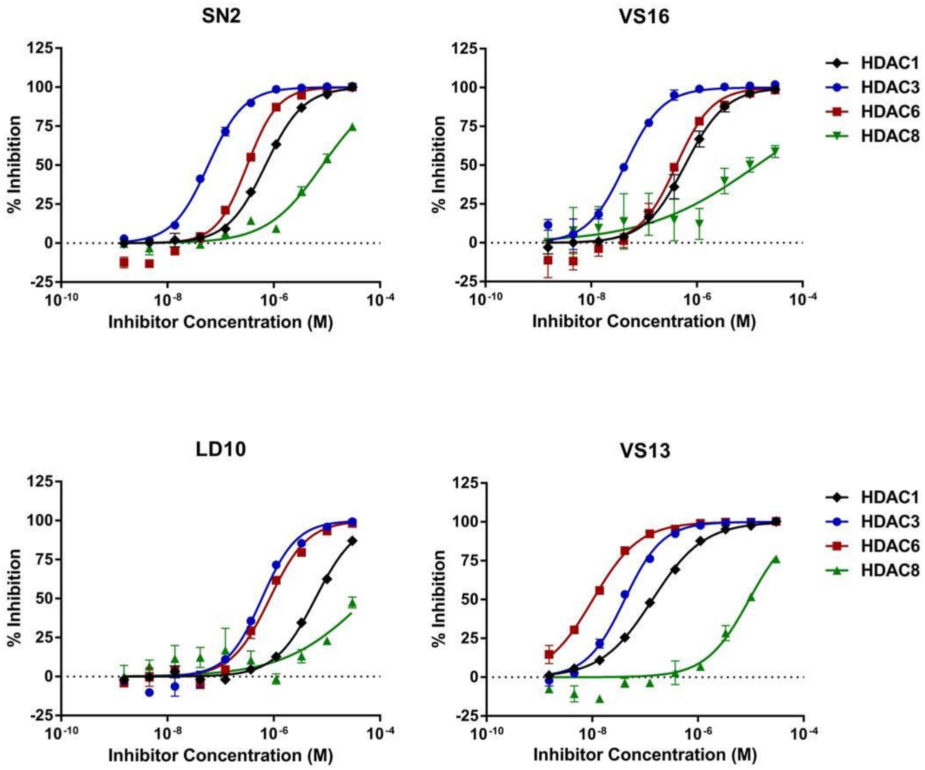

Figure S1 HDAC isoform selectivity assays. Dose-response curves for compounds **SN2**, **VS16**, **LD10** and **VS13.** Data are present as mean ± SEM. Compounds were tested in duplicate in a 10-point dose curve with 3-fold serial dilution starting from 30μM.

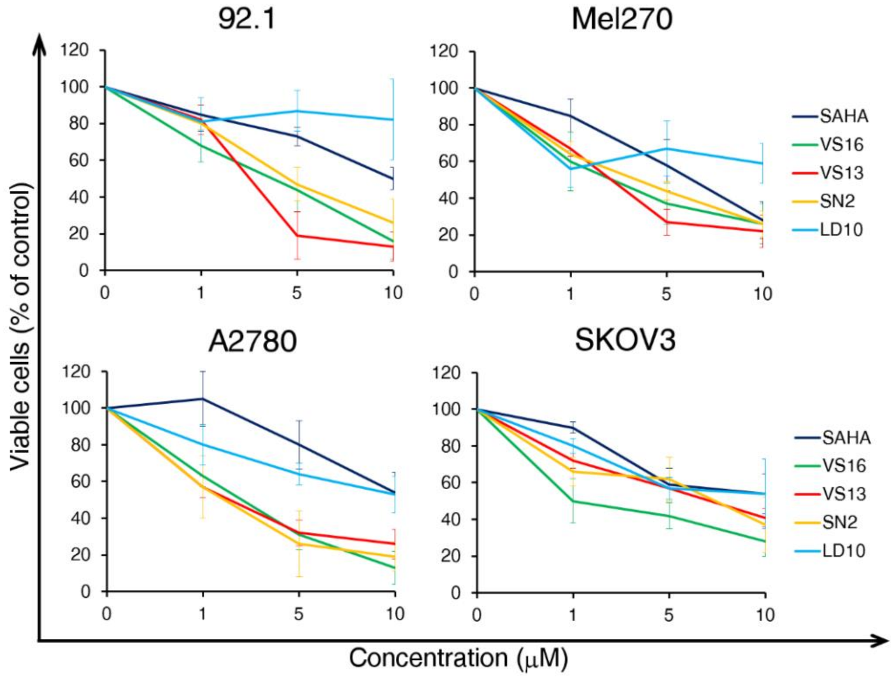

Figure S2 MTT assay. In vitro cytotoxicity at the 72 hour time point of the different compounds in human uveal melanoma cell lines 92.1 and Mel270 and ovarian cancer cell lines A2780 and SKOV3, as assessed by the MTT cell viability assay. Data are expressed as percent of control with the DMSO

solvent, which was used at the same amount present in the highest compound concentration. Error bars represent SD of quadruplicates. One representative experiment is shown.

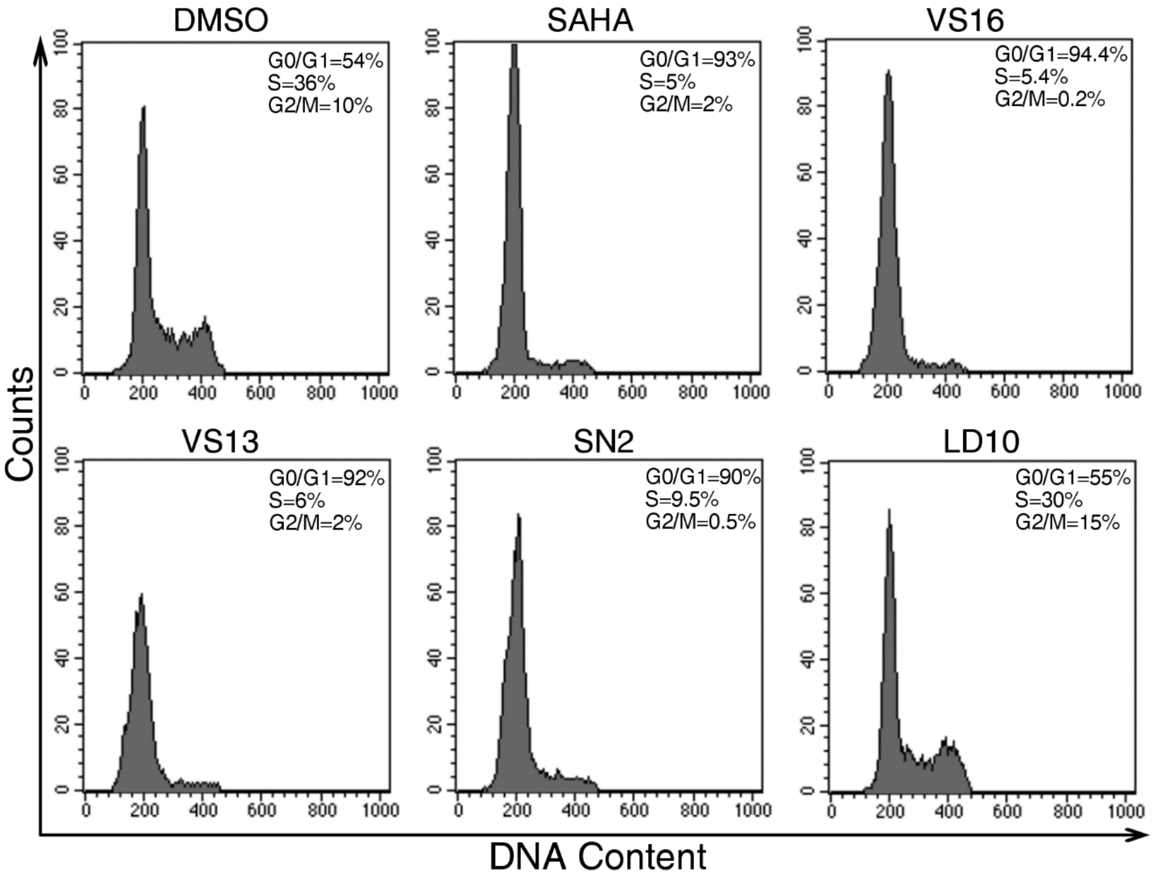

Figure S3 Cell cycle analysis. Flow-cytometric analysis of the DNA content of uveal melanoma 92.1 cells after treatment with 10 µM of the indicated compounds for 48 hours. The cells were treated and cell cycle distribution was analyzed by flow cytometry after fixation and staining with PI. The percentage of cells in each category is indicated. One representative experiment is shown.

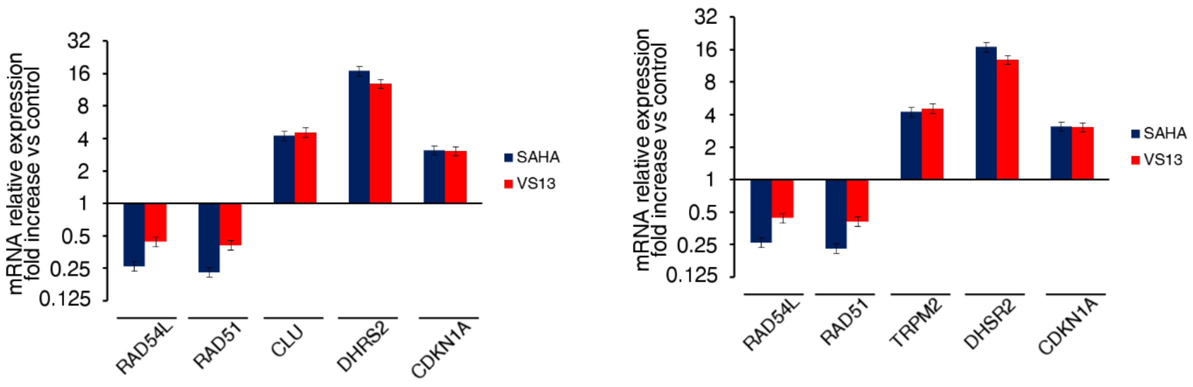

Figure S4 qRT-PCR. Comparison of the modulation of expression of five different genes induced by SAHA and VS13 in uveal melanoma cells 92.1. Cells were treated for 48 hours with 10 µM compound or the corresponding amount of DMSO. Data, normalized to GAPDH housekeeping gene, are expressed as fold change relative to the DMSO control. Error bars represent SD of triplicates. One representative experiment is shown

NMR characterization of compound **2f** (**VS13**):

^{1}H NMR (400 MHz DMSO-d_6):

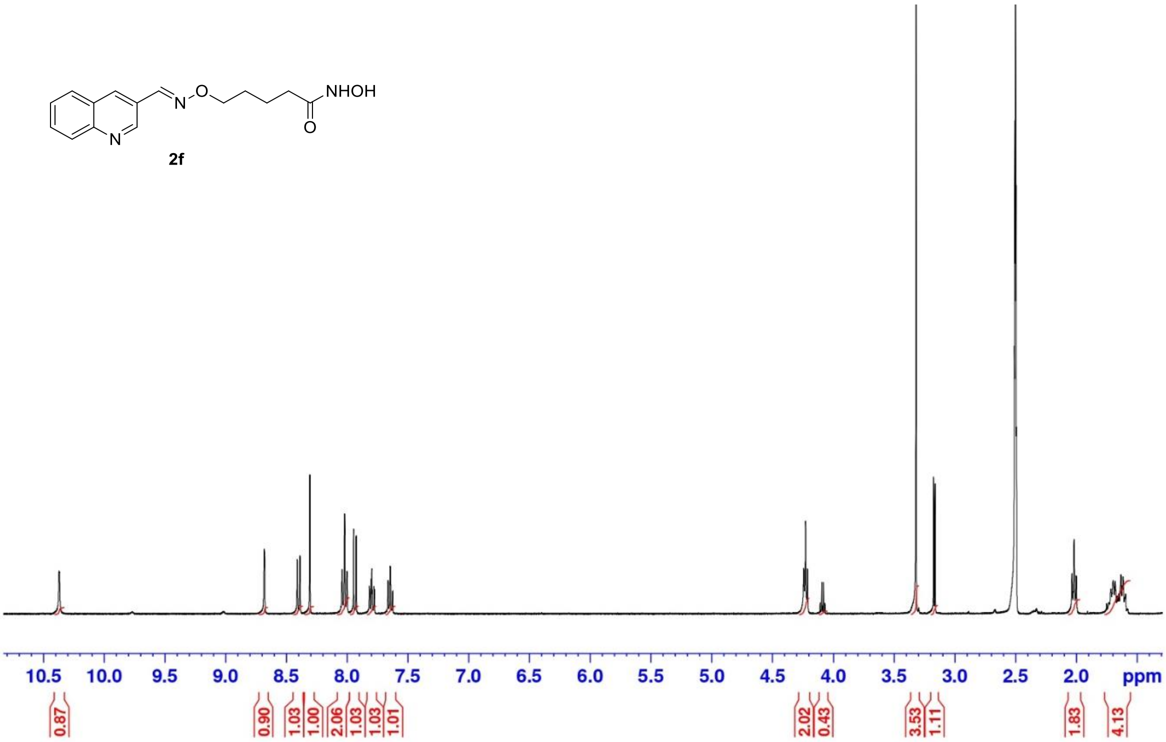

^{13}C-NMR (100 MHz DMSO-d_6):

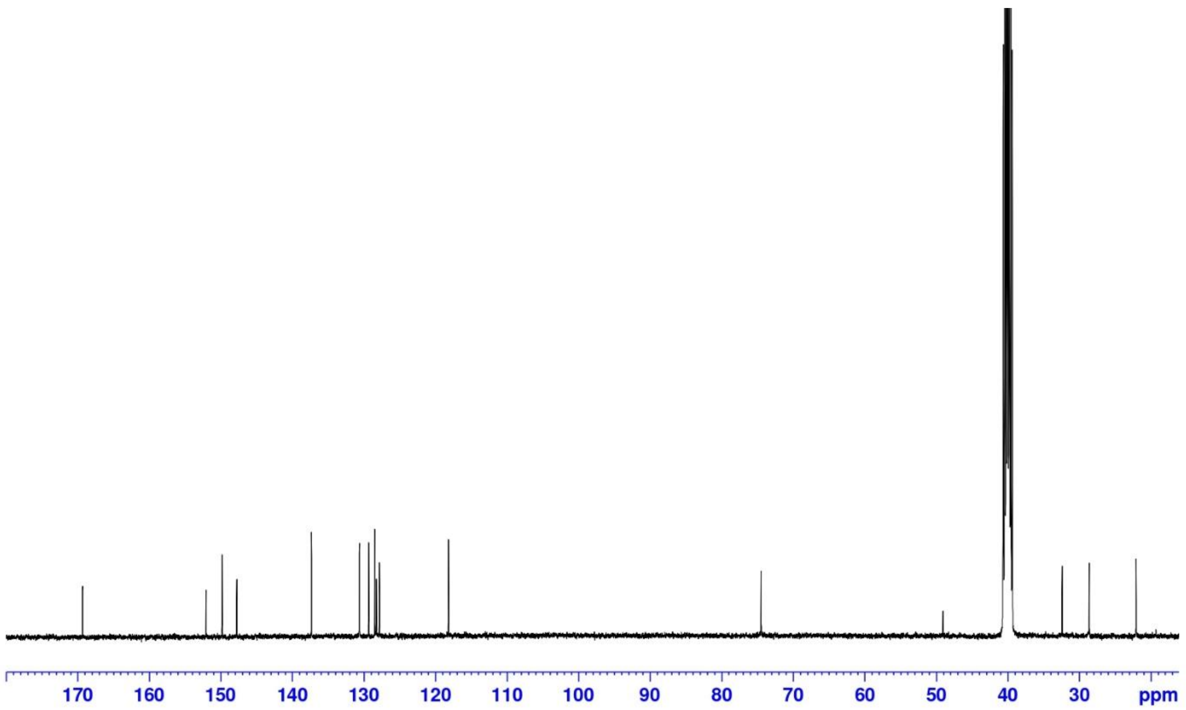
170 160 150 140 130 120 110 100 90 80 70 60 50 40 30 ppm
